# Down-regulation of WWP2 aggravates Type 2 diabetes mellitus-induced vascular endothelial injury through modulating ubiquitination and degradation of DDX3X

**DOI:** 10.1186/s12933-023-01818-3

**Published:** 2023-05-06

**Authors:** Shilong You, Jiaqi Xu, Zeyu Yin, Boquan Wu, Pengbo Wang, Mingjun Hao, Cheng Cheng, Mengke Liu, Yuanhui Zhao, Pengyu Jia, Hongkun Jiang, Da Li, Liu Cao, Xingang Zhang, Ying Zhang, Yingxian Sun, Naijin Zhang

**Affiliations:** 1 Department of Cardiology, The First Hospital of China Medical University, 155 Nanjing North Street, Heping District, Shenyang, 110001 Liaoning Province People’s Republic of China; 2 Center of Reproductive Medicine, Shengjing Hospital of China Medical University, Shenyang, 110004 China; 3 Department of Pediatrics, The First Hospital of China Medical University, 155 North Nanjing Street, Shenyang, 110001 China; 4 Key Laboratory of Reproductive and Genetic Medicine (China Medical University), National Health Commission, Shenyang, 110004 China; 5 Key Laboratory of Medical Cell Biology, Ministry of Education, 77 Puhe Road, Shenbei New District, Shenyang, 110001 Liaoning Province People’s Republic of China; 6 Institute of School of Basic Medicine, China Medical University, 77 Puhe Road, Shenbei New District, Shenyang, 110001 Liaoning Province People’s Republic of China; 7 Key Laboratory of Environmental Stress and Chronic Disease Control and Prevention, Ministry of Education, China Medical University, 77 Puhe Road, Shenbei New District, Shenyang, 110001 Liaoning Province People’s Republic of China

**Keywords:** Type 2 diabetes mellitus, Diabetic vascular complications, Endothelial injury, WWP2, DDX3X, Ubiquitination

## Abstract

**Background:**

Endothelial injury caused by Type 2 diabetes mellitus (T2DM) is considered as a mainstay in the pathophysiology of diabetic vascular complications (DVCs). However, the molecular mechanism of T2DM-induced endothelial injury remains largely unknown. Here, we found that endothelial WW domain-containing E3 ubiquitin protein ligase 2 (WWP2) act as a novel regulator for T2DM-induced vascular endothelial injury through modulating ubiquitination and degradation of DEAD-box helicase 3 X-linked (DDX3X).

**Methods:**

Single-cell transcriptome analysis was used to evaluate WWP2 expression in vascular endothelial cells of T2DM patients and healthy controls. Endothelial-specific *Wwp2* knockout mice were used to investigate the effect of WWP2 on T2DM-induced vascular endothelial injury. In vitro loss- and gain-of-function studies were performed to assess the function of WWP2 on cell proliferation and apoptosis of human umbilical vein endothelial cells. The substrate protein of WWP2 was verified using mass spectrometry, coimmunoprecipitation assays and immunofluorescence assays. The mechanism of WWP2 regulation on substrate protein was investigated by pulse-chase assay and ubiquitination assay.

**Results:**

The expression of WWP2 was significantly down-regulated in vascular endothelial cells during T2DM. Endothelial-specific *Wwp2* knockout in mice significantly aggravated T2DM-induced vascular endothelial injury and vascular remodeling after endothelial injury. Our in vitro experiments showed that WWP2 protected against endothelial injury by promoting cell proliferation and inhibiting apoptosis in ECs. Mechanically, we found that WWP2 is down-regulated in high glucose and palmitic acid (HG/PA)-induced ECs due to c-Jun N-terminal kinase (JNK) activation, and uncovered that WWP2 suppresses HG/PA-induced endothelial injury by catalyzing K63-linked polyubiquitination of DDX3X and targeting it for proteasomal degradation.

**Conclusion:**

Our studies revealed the key role of endothelial WWP2 and the fundamental importance of the JNK-WWP2-DDX3X regulatory axis in T2DM-induced vascular endothelial injury, suggesting that WWP2 may serve as a new therapeutic target for DVCs.

**Supplementary Information:**

The online version contains supplementary material available at 10.1186/s12933-023-01818-3.

## Introduction

Diabetes mellitus, especially Type 2 diabetes mellitus (T2DM), is becoming a serious public health problem and is projected to afflict over 693 million people worldwide by 2045 [1]. Diabetic vascular complications (DVCs) caused by chronic exposure to T2DM, are the leading cause of morbidity and mortality in patients with T2DM [2, 3]. Despite the successful development of new treatments for T2DM, the search for novel therapeutic targets to reduce DVCs is a continuous effort [4]. Recently, endothelial injury, which promotes the development of DVCs [5], has received much attention as a key mediator in the pathogenesis of DVCs [6–8]. However, the molecular mechanisms underlying T2DM-induced endothelial injury are largely unknown.

WW domain-containing E3 ubiquitin protein ligase 2 (WWP2), also known as atrophin-1 interacting protein 2 (AIP2), is a C2-WW-HECT-type E3 ubiquitin (Ub) ligase [9]. By regulating ubiquitination and stability of specific substrate proteins, WWP2 has emerged as a critical moderator of many biological processes including embryonic stem cell maintenance or differentiation, cartilage homeostasis, immune response, apoptosis and cell signal transduction [10–14]. As such, WWP2 injury can lead to a variety of diseases, including tumorigenesis [15], craniofacial anomalies [16], oxidative stress [17] and cardiac remodeling [18]. However, the specific role and molecular mechanism of WWP2 in DVCs are still unclear.

In this study, our single-cell analysis of endothelial cells (ECs) from T2DM patients provided insight into the involvement of WWP2 in T2DM-induced vascular endothelial injury. Subsequently, we investigated the effect of WWP2 on T2DM-induced vascular endothelial injury and vascular remodeling after endothelial injury using endothelial-specific *Wwp2* knockout mice. Also, we assessed the function of WWP2 on cell proliferation and apoptosis in vitro. Mechanically, we found that WWP2 is down-regulated in high glucose and palmitic acid (HG/PA)-induced ECs due to c-Jun N-terminal kinase (JNK) activation. Furthermore, we identified DEAD-box helicase 3 X-linked (DDX3X) as a new physiological substrate of WWP2, and found that WWP2 targets DDX3X for proteasomal degradation by catalyzing its K63-linked polyubiquitination, thereby regulating HG/PA-induced endothelial injury. Taken together, the present study revealed the crucial function of WWP2 and the fundamental importance of the JNK-WWP2-DDX3X regulatory axis in T2DM-induced vascular endothelial injury, suggesting that JNK-WWP2-DDX3X axis has potential as a preventive and therapeutic target for DVCs.

## Methods

### Single cell RNA-seq analysis

Single-cell transcriptome profiles of human mesenteric arteries from healthy and T2DM donors were derived from a public dataset GSE156341 [19]. The detailed clinical information of donors was provided in Additional file 1: Table S1. Considering the gender differences in risk, endothelial function, pathophysiology and vascular complications of T2DM [22–25], we included only male donors in the dataset GSE156341 and male mice for subsequent analysis to avoid potential variations contributed by gender and genetic background. The dataset was analyzed using the R package Seurat v.4.1.0 [26]. For quality control, count data were filtered by the following criteria: (1) removing the cells that expressing less than 200, or more than 5000 genes; (2) removing the cells with a percentage of mitochondrial genes higher than 15%; (3) removing the cells with a percentage of hemoglobin gene higher than 1%; (4) removing the cells with a percentage of ribosomal genes less than 3%; (5) removing genes that were expressed in less than 3 cells; (6) removing the MALAT1 gene. In total, the filtered data contained 6917 cells and 19,168 genes.

The filtered data were normalized and scaled using the SCTransform function [27]. The top 3000 highly variable genes of each sample were identified using SelectIntegrationFeatures function. To remove batch effect across samples, we performed the integration pipeline provided by Seurat package built on canonical correlation analysis (CCA). After linear dimensional reduction (RunPCA function), 20 principal components (PCs) were selected for graph-based semi-unsupervised clustering (resolution 0.05) and Uniform Manifold Approximation and Projection (UMAP) dimensionality reduction and visualization.

The batch effects across samples were successfully removed by CCA method (Additional file 1: Fig. S1A, B) [26] and the FindAllMarkers function was applied to identify marker genes for each cluster (Additional file 1: Table S2). Using known genetic markers, we identified a total of four clusters: cluster 0 (T cells), cluster 1 (ECs), cluster 2 (macrophages), cluster 3 (B cells) (Additional file 1: Fig. S1C, D).

Further analysis was performed on endothelial subpopulation (clusters 1) with high expression of endothelium-specific markers, such as VWF and PECAM1 (Additional file 1: Fig. S1E, F). Differential expression analysis between two conditions were conducted with the nonparametric Wilcoxon test in the Seurat package (Additional file 1: Table S3) [28]. For visualization of gene expression data, the following functions were used: DimPlot (Seurat package), DotPlot (Seurat package), FeaturePlot (Seurat package), VlnPlot (Seurat package) and FeaturePlot_scCustom (scCustomize package).

### Generation of endothelial-specific *Wwp2*-knockout mice

Endothelial-specific *Wwp2* knockout mice (Cdh5-Cre; *Wwp2*^*fl/fl*^) on a C57BL/6J background were constructed by the Shanghai Model Organisms Center, Inc (Shanghai, China). The detailed information about the construction of Cdh5-Cre; *Wwp2*^*fl/fl*^ mice was shown in (Fig. 2A). Genotyping for endothelial-specific *Wwp2*-knockout mice was performed using the following primers: Cdh5-Cre (5′-TCGATGCAACGAGTGATGAG-3′, 5′-TCCATGAGTGAACGAACCTG-3′, 5′-CAAATGTTGCTTGTCTGGTG-3′, and 5′-GTCAGTCGAGTGCACAGTTT-3′), *Wwp2*^*fl/fl*^ (5′-TGCCTATCCTTTCTGTGCC-3′, 5′-TTTGCCGCCATTGTTCTTC-3′, and 5′-TCGCCTTCTTGACGAGTTCT-3′).

### Animal treatment

Mice were housed in a pathogen-free and temperature-controlled conditions (22–25 ± 1 °C) under a 12 h light/dark cycle, with Food and water ad libitum. Eight-week-old male mice were used for experiments. Cdh5 Cre + ; *Wwp2*^*fl/fl*^ and Cdh5 Cre-; *Wwp2*^*fl/fl*^ mice were randomly assigned to two groups: T2DM group and control group (Fig. 2D). For T2DM group, the mice received a high-fat diet (HFD; MD12033, Medicience Ltd., Jiangsu, China) for 6 weeks followed by intraperitoneal injection of streptozotocin (STZ, HY-13753, MCE, USA) at a dose of 40 mg/kg after overnight fasting for 5 consecutive days. Two weeks after STZ injection, the HFD/STZ mice with fasting blood glucose level ≥ 16.7 mM were confirmed as T2DM mice and then continued to receive the HFD for 4 months; For control group, the mice were injected with the same volume of citrate buffer (0.1 M, pH 4.5) after 6 weeks of normal diet (ND), and then continued to be fed the ND until the end of the experiment. Body weight was recorded every two weeks and blood glucose levels were monitored monthly from the tail-tip with a glucometer. Before sampling, mice were fasted overnight and serum triglyceride (TG), total cholesterol (TC) measurements were measured with the commercial detection kits (Nanjing Jiancheng Bioengineering Institute, China) according to the manufacturer's instructions. This study was performed in line with the principles of the Declaration of Helsinki. The Animal Subject Committee of China Medical University approved the animal study protocol (permission number: CMU2023364).

### Immunofluorescence assays

Frozen sections of mouse aorta were fixed in acetone for 10 min at – 20 ºC, washed three times with PBS, and incubated overnight at 4 °C with primary antibodies in a blocking buffer (10% BSA, 10% Goat Serum in PBS): anti-WWP2 (Proteintech, USA), anti-DDX3X (Bethyl Laboratories, USA), anti-CD31(Santa Cruz Biotechnology, USA). After washing with PBS three times, the sections were incubated with corresponding secondary antibodies, including Donkey anti-Mouse IgG (H + L) Highly Cross-Adsorbed Secondary Antibody, Alexa Fluor™ 488 (A-21202, Invitrogen, USA) and Donkey anti-Rabbit IgG (H + L) Highly Cross-Adsorbed Secondary Antibody, Alexa Fluor™ 594 (A21207, Invitrogen, USA), for 2 h at room temperature (RT). The sections were counterstained with 4′,6-diamidino-2-phenylindole (DAPI; C0065, Solarbio, Beijing, China) and imaged with a fluorescence microscope (Nikon Eclipse 90i, Japan). To evaluate the expression level of endothelial-cell WWP2, the mean fluorescence intensity of WWP2 per pixel in the vascular endothelium region was calculated using ImageJ software version 1.46 (National Institutes of Health, USA). In addition, TUNEL staining was conducted with TUNEL Apoptosis Detection Kit (KTA2011, Abbkine, USA) following the manufacturer’s instructions. The percentage of apoptotic ECs was calculated by determining the number of TUNEL positive cells with respect to the total number of CD31 positive cells in the vascular endothelium.

For cell colocalization analysis, human umbilical vein endothelial cells (HUVECs) were fixed with 4% paraformaldehyde in PBS for 15 min, permeabilized with 0.5% triton X-100 in PBS for 10 min and blocked with 5% goat serum for 1 h at RT. Cells were incubated in primary antibody overnight at 4 °C (anti-WWP2, 1:100, Proteintech, USA; anti-DDX3X, 1:100, Santa Cruz Biotechnology, USA), and in secondary antibody for 2 h at RT [Donkey anti-Mouse IgG (H + L) Highly Cross-Adsorbed Secondary Antibody, Alexa Fluor™ 488, 1:250, Invitrogen, USA; Donkey anti-Rabbit IgG (H + L) Highly Cross-Adsorbed Secondary Antibody, Alexa Fluor™ 594, 1:250, Invitrogen, USA]. After antibody treatment, HUVECs were stained with DAPI solution (C0065, Solarbio, Beijing, China) to mark nuclei and were observed under Nikon A1 confocal microscope. Colocalization and quantification analysis was performed using the Plot Profile function and Coloc 2 plugin of Fiji ImageJ software.

### Histological analysis

Formalin-fixed, Paraffin-embedded aortic tissues were sectioned and stained with hematoxylin and eosin (H&E) and a Masson's Trichrome Stain Kit (G1340, Solarbio, Beijing, China), as described previously [20]. Images were captured with a microscope (FLEXACAM C1, Leica). Quantitative analysis of vascular wall thickness and vascular fibrosis were implemented by ImageJ version 1.46 (National Institutes of Health, USA).

### Cell culture and treatment

HUVEC line was purchased from ATCC and cultured in F‐12K medium (Procell, Wuhan, China) with 10% fetal bovine serum (FBS; HyClone, USA), 1% penicillin/streptomycin (Biosharp, Heifei, China), heparin (0.1 mg/mL) and endothelial cell growth supplement (ECGs, 0.05 mg/mL). Cells were maintained at 37 ºC in a humid environment with 5% CO_2_ and used between passages 4–6 for experiments. HUVECs were stimulated with HG (33 mM)/PA (100 μM; HY-N0830, MCE, USA) for 48 h. Mannitol [MAN, 33 mM: 5.5 mM of glucose + 27.5 mM of D-mannitol (M4125, Sigma-Aldrich, USA)] was used as the osmotic control for the HG/PA. JNK inhibitor SP600125 (20 μM, 24 h; S1460, Selleck, USA) and JNK activator Anisomycin (10 μM, 24 h; S7409, Selleck, USA) were used to inhibit and induce JNK activation in HUVECs, respectively. For the protein stability and degradation assay, HUVECs were treated with cycloheximide (CHX, 20 μM; A8244, ApexBio, USA) for indicated duration or proteasome inhibitor MG132 (10 μM; A2585, ApexBio, USA) for 6 h, or lysosome inhibitor chloroquine (CQ, 50 μΜ; Sigma-Aldrich, USA) for 24 h. Each experimental result was repeated for three independent biological replicates.

### Quantitative real-time PCR

Total RNA was extracted from the HUVECs with TRIzol Reagent (Invitrogen, USA). Reverse transcription was performed by the PrimeScript RT reagent Kit with gDNA Eraser (Takara, RR047A). Real-time polymerase chain reaction (PCR) was performed in triplicate for each sample and each gene using Applied Biosystems 7500 Fast Real-Time PCR System, and the average cycle thresholds (Ct) values were recorded in this analysis system. The mRNA expression were calculated by the 2 ^−△△Ct^ method using GAPDH as an endogenous control. The primers designed from Sangon Biotech Co., Ltd (Shanghai, China) are as follows: WWP2 (forward primer: 5′-GAGATGGACAACGAGAAG-3′; reverse primer: 5′-CTCCTCAATGGCATACAG-3′), GAPDH (forward primer: 5′-CTGGGCTACACTGAGCACC-3′; reverse primer: 5′-AAGTGGTCGTTGAGGGCAATG-3′).

### Plasmids, siRNAs and cell transfections

A HA-tagged human full-length WWP2 expression Plasmid was obtained from Genechem (Shanghai, China). Ub and Ub mutants, including K48 (only K48-linked Ub) and K63 (only K63-linked Ub), were gifts of zhou tingting (Department of Cell Biology, Key laboratory of Cell Biology, Ministry of Public Health, and Key laboratory of Medical Cell Biology, Ministry of Education, School of Life Sciences, China Medical University, Shenyang, Liaoning, China). WWP2 small interfering RNAs (siRNAs) and negative controls were purchased from RIBOBIO (Guangzhou, China). Three siRNAs targeting human WWP2 were designed to avoid off-target effects and successful knockdown of WWP2 was confirmed by western blotting (Fig. 7B):

WWP2 siRNA-1: GATCTGGGAAATGTGCCTA.

WWP2 siRNA-2: GGTGCTTCAGCCAGAACAA.

WWP2 siRNA-3: CGGACGTGTCTATTATGTT.

DDX3X siRNAs and negative controls were also purchased from RIBOBIO (Guangzhou, China), and the three DDX3X siRNAs targeting human DDX3X are as follows:

DDX3X siRNA-1: GATCTGGGAAATGTGCCTA.

DDX3X siRNA-2: GGTGCTTCAGCCAGAACAA.

DDX3X siRNA-3: CGGACGTGTCTATTATGTT.

Successful knockdown of DDX3X was confirmed by western blotting (Fig. 8A, B). The plasmid or siRNA was transfected with Lipofectamine 3000 (Invitrogen, USA) or jetPRIME (Polyplus, France), respectively, according to the manufacturer's protocol. For single transfections, HUVECs were grown to 70–80% confluence and transfected with control or HA-WWP2 plasmids (5 μg for 6-cm dish; 10 μg for 10-cm dish) using Lipofectamine 3000 (plasmid/transfection reagent = 1 μg/2.4 μL) or control-siRNA or WWP2 siRNAs (final concentration 20 nM) using jetPRIME (siRNA/transfection reagent = 20 pmol/μL). For co-transfection experiments, HUVECs were grown to 70–80% confluence and co-transfected with control or HA-WWP2 plasmids (8 μg for 10-cm dish) and Ub or Ub mutants plasmids (6 μg for 10-cm dish) using Lipofectamine 3000 (plasmid/transfection reagent = 1 μg/2.4 μL). The medium was replaced with fresh normal medium 6–8 h (plasmid) or 12 h (siRNA) after transfection. HUVECs were harvested 48–72 h post-transfection.

### Western blotting

Western blotting was performed using the protocol previously described [29]. Briefly, HUVECs or vascular tissues were lysed in lysis buffer (50 mM Tris, pH 7.8, 137 mM NaCl, 1 mM EDTA, 10 mM NaF, 0.1 mM Na3VO4, 1% NP-40, 10% glycerol, with protease inhibitors) on the ice for 30 min. Protein samples were collected by centrifugation at 13,000 rpm at 4 °C for 20 min, separated by SDS–PAGE and transferred to PVDF membranes (Millipore, USA). The membranes were blocked for 1 h with 5% nonfat milk in TBST buffer (20 mM Tris, pH 7.4, 137 mM NaCl and 0.05% Tween‑20), and then incubated with specific primary antibodies overnight at 4 °C. Antibodies were used as follows: anti-WWP2 (1:1000, Proteintech, USA, #12197-1-AP), anti-WWP2 (1:1000, abcam, UK, #ab103527), anti-DDX3X (1:1000, Bethyl Laboratories, USA, #A300-474A), anti-DDX3X (1:500, Santa Cruz Biotechnology, USA, #sc-365768), anti-Cleaved PARP1 (1:1000, Cell Signaling Technology, USA, #9541S), anti-Cleaved Caspase-3 (1:1000, Cell Signaling Technology, USA, #9664S), anti-HA (1:1000; Cell Signaling Technology, USA, #3724S), anti-Collagen I (1:500, Santa Cruz Biotechnology, USA, #sc-293182), anti-α-SMA (1:2000; Proteintech, USA, #23660-1-AP), anti-p-JNK (1:1000 Santa Cruz Biotechnology, USA, #sc-6254), anti-α tublin (1:2000, Proteintech, USA, #11224-1-AP). After washing with TBST three times, the membranes were incubated with second antibodies for 2 h at RT. Finally, protein bands were visualized by chemiluminescence (Tanon Science & Technology Co., Ltd., Shanghai, China) and quantified using ImageJ software version 1.46 (National Institutes of Health, USA).

### In vitro cell proliferation and apoptosis analysis

The proliferation and viability of HUVECs was measured by a Microplate ReaderBio-Rad microplate reader (Model 680; Bio Rad Laboratories, Inc., Hercules, CA, USA) at 450 nm using the Cell Counting Kit-8 (CCK-8, Dojindo, Kumamoto, Japan), following the manufacturer's instructions.

Cell apoptosis was analyzed by flow cytometry using the Annexin V-FITC Apoptosis Detection Kit (dojindo laboratories, Kumamoto, Japan) according to the manufacturer’s instruction. Flow cytometry was performed using the LSRFortessa (BD) and flow data was analyzed using FlowJo v.10 (Tree Star).

### Co-immunoprecipitation

HUVECs were washed with PBS after treatment and lysed in the above lysis buffer on ice for 45 min. The lysates were cleared by centrifugation at 13,000 rpm for 20 min at 4 °C, and incubated with anti-WWP2 antibodies (abcam; UK) or anti-DDX3X antibodies (Santa Cruz Biotechnology, USA) for 3 h, followed by incubation with 20 ul of protein A/G agarose beads (Santa Cruz Biotechnology, USA) for further 12 h at 4 °C. The immune complexes were then washed three times with lysis buffer and analysed by western blotting as described. Protein bands were visualized by chemiluminescence (Tanon Science & Technology Co., Ltd., Shanghai, China) and quantified using ImageJ software version 1.46 (National Institutes of Health, USA).

### Ubiquitylation assays

Forty-eight hours after transfection, HUVECs were lysed and whole cell lysates were incubated overnight with anti-DDX3X antibody (Santa Cruz Biotechnology, USA) and protein A/G agarose beads (Santa Cruz Biotechnology, USA) at 4 °C. The proteins were then released from the beads by boiling in 30 μL of 2 × SDS-PAGE sample buffer for 10 min and analysed by western blotting with anti-HA (Cell Signaling Technology, USA, #3724S) or anti-Ub (Cell Signaling Technology, USA, #3936S).

### Statistical analysis

All quantitative data are shown as the mean ± SEM. Statistical comparison between two experimental groups was performed using two-tailed unpaired Student t-tests. Statistical differences among more than two groups were analyzed by one-way ANOVA with Dunnett’s multiple comparison post-hoc test or Tukey’s multiple comparison post-hoc test, and two-way ANOVA with Bonferroni’s multiple comparison post hoc test. All statistical analysis were performed with Prism 8 (GraphPad, La Jolla, CA) and SPSS (Version 25). Statistical significance level was set as follows: *P < 0.05, **P < 0.01, ***P < 0.001, or NS = not significant (P > 0.05).

## Results

### Characterization of WWP2 expression in vascular endothelial cells during T2DM

**Fig. 1:**
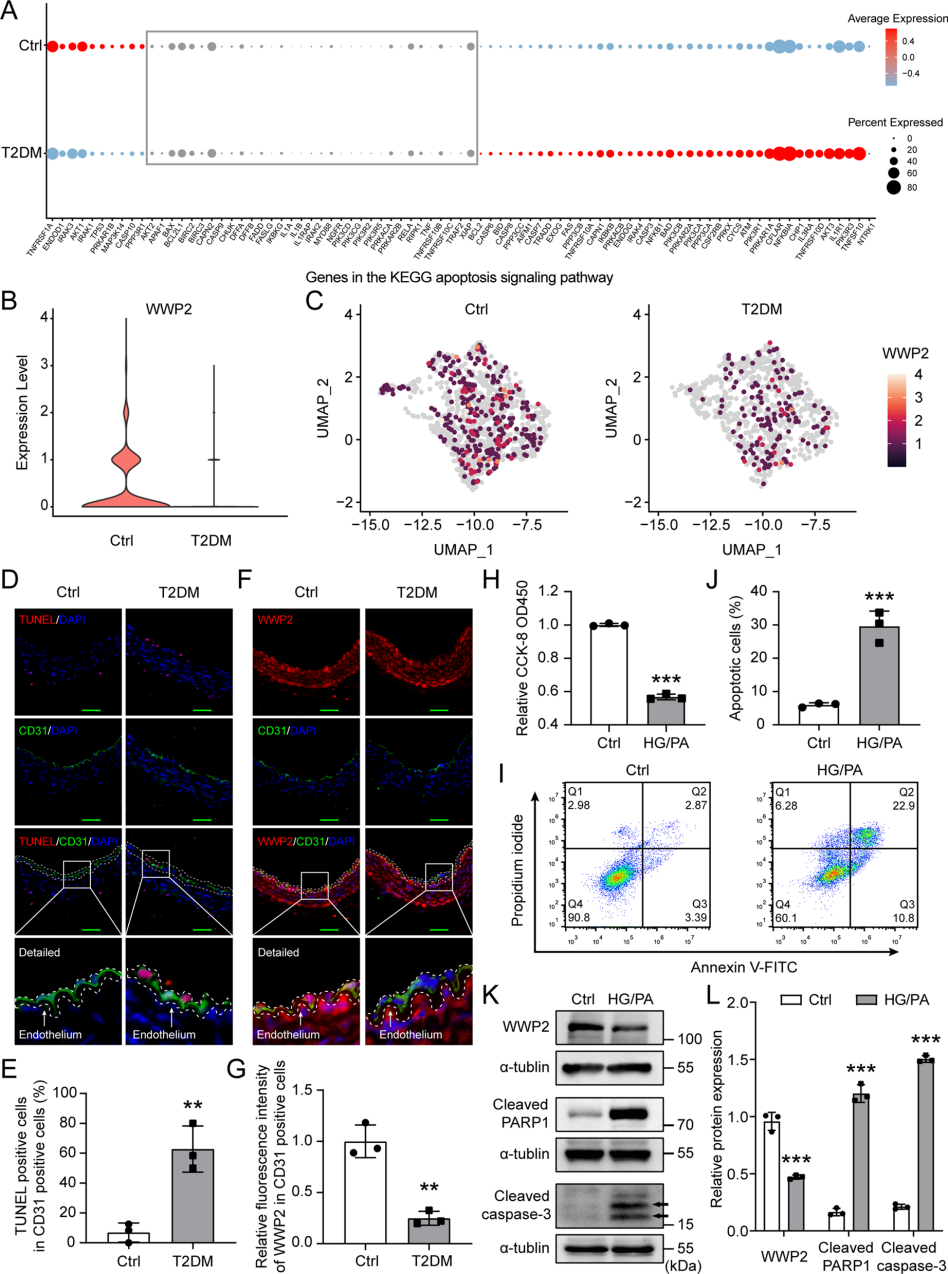
The expression levels of WWP2 in vascular endothelial cells decline during T2DM. **A** Dot plot showing expression levels of genes in the KEGG apoptosis pathway in ECs of healthy control and T2DM donors (grey represent not significant). A total of 1,939 ECs were extracted from mesenteric arterial cells from 2 healthy control and 1 T2DM male donors. **B** Violin plots showing gene expression levels of WWP2 in ECs of T2DM patients and healthy controls. **C** Feature plots showing the percentage of cells expressing WWP2 in ECs of T2DM patients (28.1%) and healthy controls (17.3%). **D** Representative co-immunofluorescent staining for TUNEL (red; a marker of apoptosis) and CD31 (green; a marker of ECs) in aortic cross sections from control or T2DM wild-type mice (n = 3 mice per group). scale bar 50 µm. **E** Quantification of apoptotic ECs in CD31 positive cells. **F** Representative co-immunofluorescent staining for WWP2 (red) and CD31 (green) in aortic cross sections from control or T2DM wild-type mice (n = 3 mice per group). scale bar 50 µm. **G** Quantitative analysis of relative fluorescence intensity of WWP2 in CD31 positive cells. **H** Cell Counting Kit-8 colorimetric assay was used to evaluate the effects of HG/PA treatment on the proliferation and viability of HUVECs. **I, J** Representative Flow cytometry analysis (**I**) and quantitative analysis (**J**) of HUVECs apoptosis induced by HG/PA. **K, L** Representative Western blots (**K**) and quantification (**L**) of WWP2, Cleaved PARP1 and Cleaved caspase-3 protein expression levels in HUVECs with or without HG/PA treatment. Values are shown as mean ± SD (**P < 0.01, ***P < 0.001, two-tailed unpaired Student t-tests). Ctrl, control; DAPI, 4′,6-diamidino-2-phenylindole; ECs, Endothelial cells; HG/PA, high glucose/palmitic acid; HUVECs, HUVECs, Human umbilical vein endothelial cells; KEGG, Kyoto Encyclopedia of Genes and Genomes; T2DM, Type 2 diabetes mellitus

To examine whether endothelial injury is related to the pathogenesis of DVCs caused by chronic T2DM, we evaluated the expression of apoptosis-related genes in vascular endothelial cells in patients with T2DM and healthy controls by single-cell analysis. We found most of the pro-apoptotic genes in apoptosis signaling pathway were up-regulated in the vascular endothelial cells of T2DM patients compared to healthy controls (Fig. 1A) (Additional file 1: Table S3), suggesting that T2DM-induced vascular endothelial injury is a key event in the development of DVCs. To decipher the mechanism of T2DM-induced vascular endothelial injury, we investigated the gene expression of WWP2, a vital E3 Ub ligase in cells that regulates various biological processes such as apoptosis [14] and maintenance of vascular integrity [30]. We observed that the expression level of WWP2 (Fig. 1B) and the percentage of cells expressing WWP2 (Fig. 1C) were reduced in ECs of T2DM patients compared to healthy controls, suggesting that the downregulation of WWP2 may be associated with T2DM-induced vascular endothelial injury.

T2DM is characterized by hyperglycemia, elevated circulating lipid levels, and insulin resistance [31]. Evidences suggest that glucotoxicity and lipotoxicity occurred in T2DM do not act alone, but are interrelated and synergistic in their adverse effects on cells or tissues [32–34]. In addition, STZ induces hyperglycemia leading to endothelial injury due to its specific toxicity to mouse pancreatic beta cells [35]. Therefore, we adopted HFD/STZ-induced mice as animal models of T2DM [36, 37], and HG/PA-induced HUVECs as in vitro models [33, 38], to simulate endothelial injury induced by glucolipotoxicity of T2DM. Immunofluorescence staining showed that the endothelial apoptosis was significantly increased (Fig. 1D, E) and the endothelial WWP2 expression was substantially decreased (Fig. 1F, G) in T2DM mice compared with wild-type control mice. Our in vitro results showed that treatment of HG/PA substantially inhibited cell proliferation and induced apoptosis, as evidenced by CCK-8 colorimetric assay (Fig. 1H) and flow cytometry analysis (Fig. 1I, J). Furthermore, Western blotting revealed that treatment of HG/PA significantly increased the protein levels of apoptotic markers cleaved PARP1 and cleaved caspase-3, and substantially decreased the protein levels of WWP2 in HUVECs (Fig. 1K, L). Taken together, these observations suggest that WWP2 is involved in the regulation of T2DM-induced vascular endothelial injury.

### Endothelial-specific *Wwp2* knockout in mice aggravates T2DM-induced vascular endothelial injury and vascular remodeling after endothelial injury

**Fig. 2:**
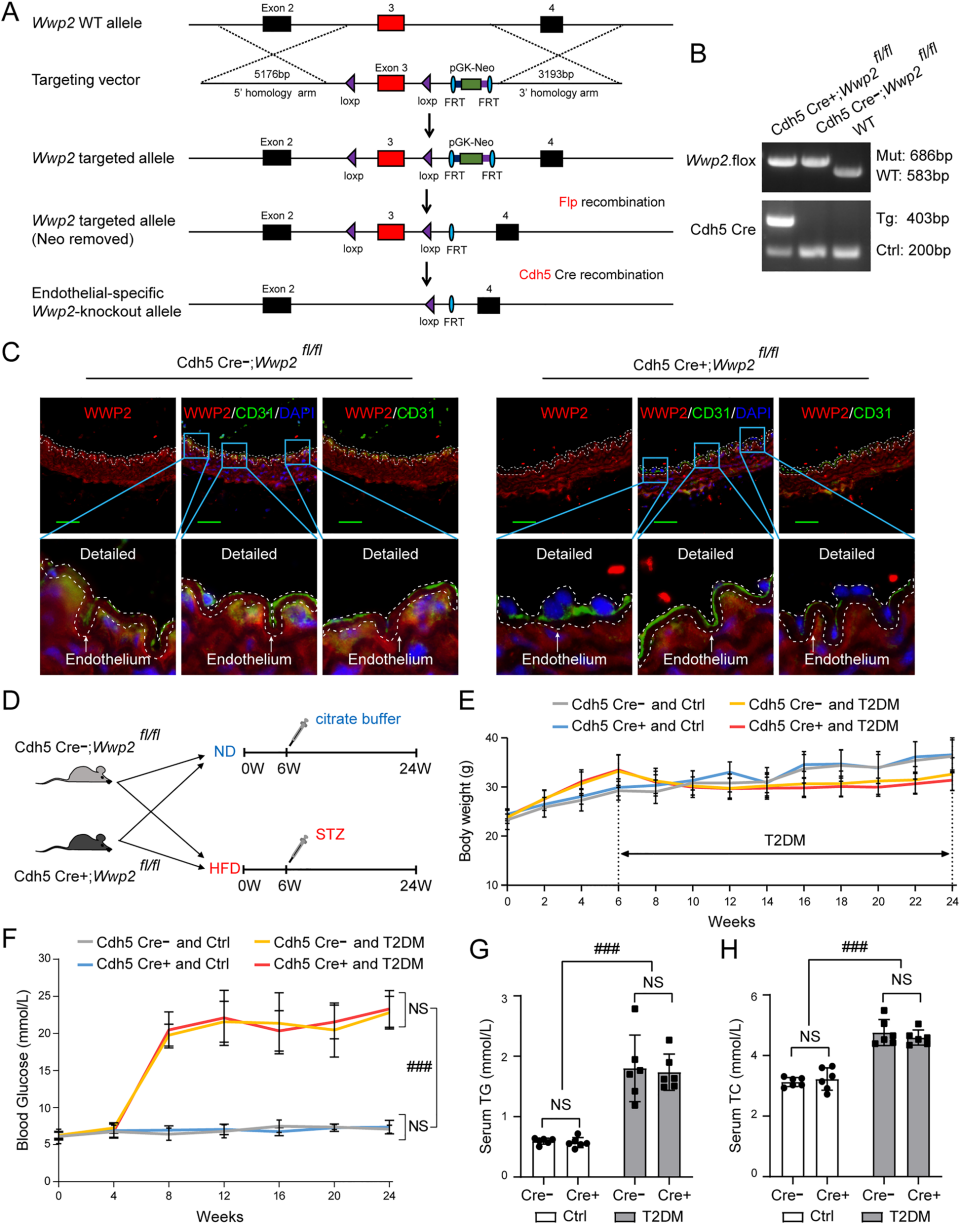
Generation of endothelial-specific *Wwp2* knockout mice and construction of a T2DM mouse model. **A** Schematic diagram of the construction of endothelial-specific *Wwp2* knockout (Cdh5 Cre + ; *Wwp2*^*fl/fl*^) mice. **B** Representative PCR image of tail genotyping of each group. **C** Co-immunofluorescent staining for WWP2 and CD31 was used to detect the expression of WWP2 in vascular endothelium of Cdh5 Cre-; *Wwp2*^*fl/fl*^ and Cdh5 Cre + ; *Wwp2*^*fl/fl*^ mice. Red, WWP2; Green, CD31 (an endothelial marker); blue, DAPI. scale bar 50 µm. **D** Schematic illustration of the construction of the mouse model for T2DM. **E** Body weights for every mouse were recorded biweekly. **F** Blood glucose levels for every mouse were measured monthly. **G**, **H** The serum TG (**G**), TC (**H**) levels were estimated at the end of the experiment. Values are shown as mean ± SD (**P < 0.01, ***P < 0.001, ###P < 0.001, NS = not significant, two-way ANOVA with Bonferroni’s multiple comparison post hoc test). Cdh5 Cre-, (Cdh5 Cre-; *Wwp2*^*fl/fl*^ mice); Cdh5 Cre + , (Cdh5 Cre + ; *Wwp2*^*fl/fl*^ mice); Ctrl, control; DAPI, 4′,6-diamidino-2-phenylindole; ECs, Endothelial cells; HFD, high-fat diet; ND, normal diet; STZ, streptozotocin; T2DM, Type 2 diabetes mellitus; TC, total cholesterol; TG, triglyceride; WT, wild-type

To further investigate the role of WWP2 in T2DM-induced vascular endothelial injury, endothelial-specific *Wwp2* knockout mice (Cdh5 Cre + ; *Wwp2*^*fl/fl*^ mice) were generated. Cdh5 Cre + ; *Wwp2*^*fl/fl*^ mice were generated by Cdh5-Cre [39] mediated deletion of floxed *Wwp2* allele in mice (Fig. 2A). Genotype of mice were identified by PCR Amplification of tail DNA (Fig. 2B). Endothelial-specific *Wwp2* deletion was confirmed by immunofluorescent staining (Fig. 2C). Then, we compared the responses of Cdh5 Cre-; *Wwp2*^*fl/fl*^ and Cdh5 Cre + ; *Wwp2*^*fl/fl*^ mice with T2DM (Fig. 2D). T2DM mice gained weight faster than controls before STZ injection, whereas the reverse was observed after STZ injection (Fig. 2E). As shown in Fig. 2F, the dynamic monitoring of blood glucose levels showed that the blood glucose of T2DM mice was ≥ 16.7 mM after STZ injection, and the blood glucose of control mice was within the normal range during the whole study period. Moreover, Serum TC and TG levels of all groups of T2DM mice were significantly higher than those of all groups of control mice, indicating that T2DM model was successfully established (Fig. 2G, H).

**Fig. 3:**
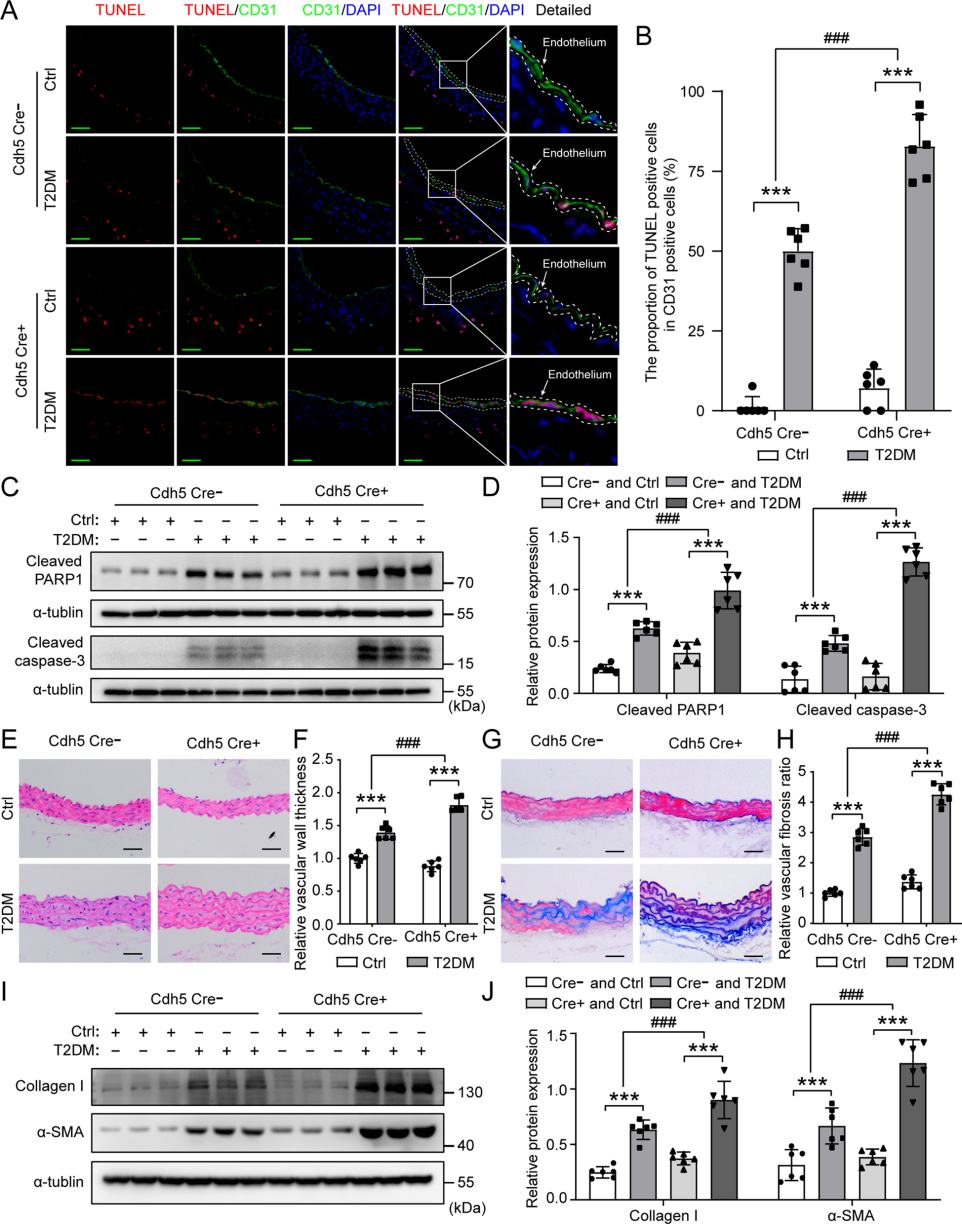
Endothelial-specific *Wwp2* knockout in mice aggravates T2DM-induced vascular endothelial injury and vascular remodeling after endothelial injury. **A**, **B** Representative immunofluorescent staining of aortic cross sections for each group (**A**) and quantification of apoptotic ECs (**B**) (n = 6 mice per group). Red, TUNEL (a marker of apoptosis); Green, CD31 (a marker of ECs); blue, DAPI. scale bar 50 µm. **C, D** Representative Western blots (**C**) and quantification (**D**) of Cleaved PARP1 and Cleaved caspase-3 protein levels in each group (n = 6 mice per group). Total protein was extracted from vascular tissue and analyzed by Western blotting. **E, F** Representative microscopy images of H&E staining of aortic cross sections (**E**) and quantification of vascular wall thickness (**F**) for each group (n = 6 mice per group). Scale bars: 50 µm. **G, H** Representative microscopy images of Masson’s trichrome staining of aortic cross sections (**G**) and quantification of vascular fibrosis (**H**) for each group (n = 6 mice per group). Scale bars: 50 µm. **I, J** Representative Western blots (**I**) and quantification (**J**) of Collagen I and α-SMA protein levels in each group (n = 6 mice per group). Total protein was extracted from vascular tissue and analyzed by Western blotting. Values are shown as mean ± SD (***P < 0.001, ###P < 0.001, two-way ANOVA with Bonferroni’s multiple comparison post hoc test). Cdh5 Cre-, (Cdh5 Cre-; *Wwp2*^*fl/fl*^ mice); Cdh5 Cre + , (Cdh5 Cre + ; *Wwp2*^*fl/fl*^ mice); Ctrl, control; DAPI, 4′,6-diamidino-2-phenylindole; ECs, Endothelial cells; H&E, hematoxylin and eosin; T2DM, Type 2 diabetes mellitus

Next, we evaluated the impact of *Wwp2* deficiency on T2DM induced apoptosis of vascular endothelial cells by co-immunofluorescent staining for TUNEL (red) and CD31 (green). Compared with Cdh5 Cre-; *Wwp2*^*fl/fl*^ mice, Cdh5 Cre + ; *Wwp2*^*fl/fl*^ mice exhibited increased apoptosis in vascular endothelial cells induced by T2DM (Fig. 3A, B). In addition, the expression levels of cleaved PARP1 and cleaved caspase-3, which are markers of apoptosis, were markedly elevated in T2DM-induced Cdh5 Cre + ; *Wwp2*^*fl/fl*^ mice compared to T2DM-induced Cdh5 Cre-; *Wwp2*^*fl/fl*^ mice (Fig. 3C, D). Consequently, endothelial-specific loss of *Wwp2* exacerbates T2DM-induced vascular endothelial injury.

Diabetes-induced endothelial injury leads to vascular remodeling, including vascular thickening and fibrosis, which contribute to the development of DVCs [40, 41]. Therefore, we assessed the effect of endothelial-specific *Wwp2* loss on vascular thickening and fibrosis induced by T2DM. In comparison with Cdh5 Cre-; *Wwp2*^*fl/fl*^ mice, Cdh5 Cre + ; *Wwp2*^*fl/fl*^ mice exhibited increased vascular wall thickness (Fig. 3E, F). Masson’s trichrome staining revealed larger areas of vascular fibrosis in Cdh5 Cre + ; *Wwp2*^*fl/fl*^ mice (Fig. 3G, H). In addition, Cdh5 Cre + ; *Wwp2*^*fl/fl*^ mice had significantly elevated levels of markers of fibrosis, Collagen I and α-SMA, as compared with Cdh5 Cre-; *Wwp2*^*fl/fl*^ mice (Fig. 3I, J). Together, these data confirm that endothelial-specific *Wwp2* knockout in mice significantly aggravates T2DM-induced vascular endothelial injury and vascular remodeling after endothelial injury.

### WWP2 protects against HG/PA-induced endothelial injury in vitro

**Fig. 4:**
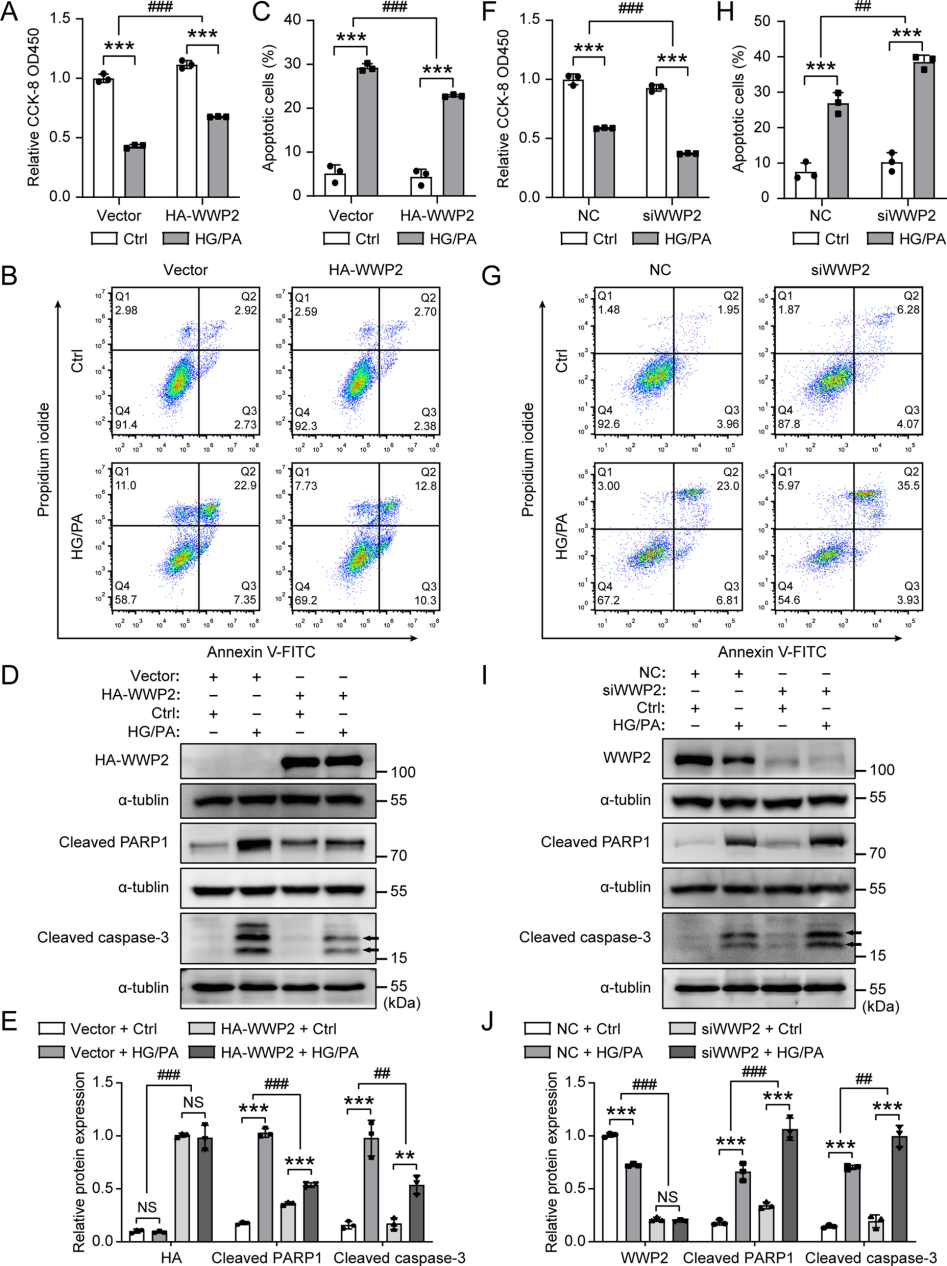
WWP2 inhibits endothelial injury in vitro. **A**, **F** The proliferation and viability of HUVECs was tested using the CCK-8 colorimetric assay. HUVECs were transfected with HA-WWP2 plasmid (**A**) or WWP2 siRNA (**F**) and treated with or without HG/PA for 48 h. **B, C, G, H** Representative Flow cytometry analysis (**B, G**) and quantitative analysis (**C, H**) of apoptosis in HUVECs transfected with HA-WWP2 plasmid (**B, C**) or WWP2 siRNA (**G, H**) and treated with or without HG/PA for 48 h. **D, E, I, J** Representative Western blots (**D, I**) and quantification (**E, J**) of HA-WWP2, WWP2, Cleaved PARP1 and Cleaved caspase-3 protein expression levels in vitro. HUVECs were transfected with HA-WWP2 plasmid (**D, E**) or WWP2 siRNA (**I, J**) and treated with or without HG/PA for 48 h. Values are shown as mean ± SD (**P < 0.01, ***P < 0.001, ##P < 0.01, ###P < 0.001, NS = not significant, two-way ANOVA with Bonferroni’s multiple comparison post hoc test). CCK-8, Cell Counting Kit-8; Ctrl, control; HG/PA, high glucose/palmitic acid; HUVECs, HUVECs, Human umbilical vein endothelial cells; NC, negative control; siRNA, small interfering RNA

To assess the function of WWP2 on endothelial injury in vitro, HUVECs were transfected with plasmid expressing HA-tagged human WWP2 and siRNA targeting WWP2, with or without HG/PA treatment. Using an independent sensitive colorimetric assay, we found that overexpression of WWP2 restored HG/PA-inhibited cell proliferation and viability (Fig. 4A), whereas knockdown of WWP2 had the opposite effect in HUVECs (Fig. 4F). Flow cytometry analysis showed that overexpression of WWP2 significantly alleviated HG/PA-induced HUVECs apoptosis (Fig. 4B, C), while knockdown of WWP2 significantly aggravated HG/PA-induced HUVECs apoptosis (Fig. 4G, H). Besides, overexpression of WWP2 markedly decreased the protein levels of apoptosis marker cleaved PARP1 and cleaved caspase-3 (Fig. 4D, E), while knockdown of WWP2 markedly increased the protein levels of cleaved PARP1 and cleaved caspase-3 (Fig. 4I, J). These results indicte that WWP2 protects against endothelial injury in vitro by promoting endothelial cell survival and inhibiting apoptosis.

### WWP2 is down-regulated in HG/PA-induced HUVECs due to JNK activation

**Fig. 5:**
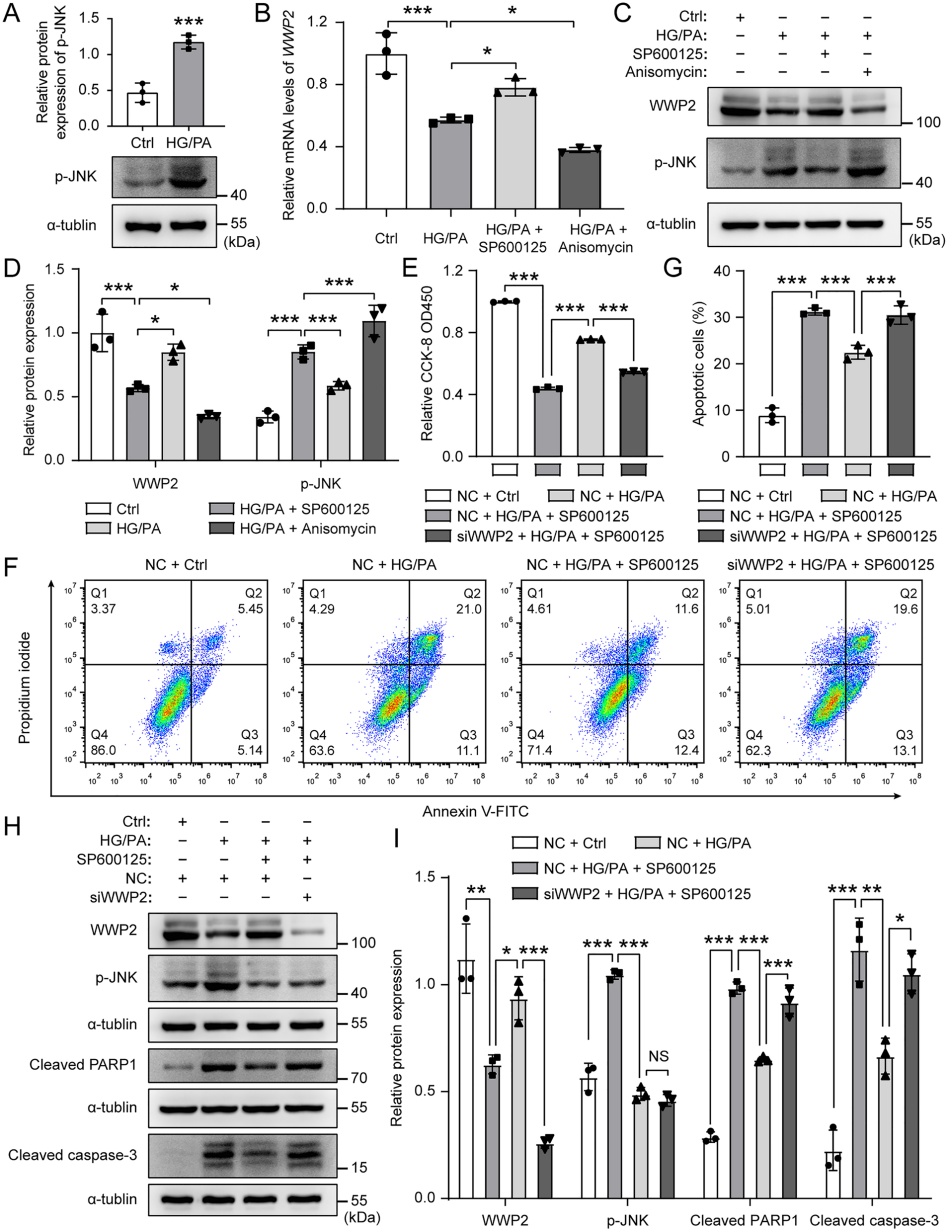
HG/PA down-regulates WWP2 through JNK activation. **A** Representative Western blots and quantification of p-JNK protein expression levels in HUVECs with or without HG/PA treatment. Values are shown as mean ± SD (**P < 0.01, ***P < 0.001, two-tailed unpaired Student t-tests). **B** Relative mRNA levels of WWP2 in HUVECs treated with JNK inhibitor (SP600125) or JNK activator (Anisomycin), with or without HG/PA. **C, D** Representative Western blots (**C**) and quantification (**D**) of WWP2 and p-JNK protein expression levels in HUVECs treated with JNK inhibitor (SP600125) or JNK activator (Anisomycin), with or without HG/PA. **E** The proliferation and viability of HUVECs was tested using the CCK-8 colorimetric assay. HUVECs were transfected with WWP2 siRNA or a negative control, and treated with JNK inhibitor (SP600125), with or without HG/PA. **F, G** Representative Flow cytometry analysis (**F**) and quantitative analysis (**G**) of apoptosis in HUVECs. **H, I** Representative Western blots (**H**) and quantification (**I**) of WWP2, p-JNK, Cleaved PARP1 and Cleaved caspase-3 protein expression levels in HUVECs. HUVECs were transfected with WWP2 siRNA or a negative control, and treated with JNK inhibitor (SP600125), with or without HG/PA. Values are shown as mean ± SD (*P < 0.05, **P < 0.01, ***P < 0.001, NS = not significant, one-way ANOVA with Tukey’s multiple comparison post-hoc test). CCK-8, Cell Counting Kit-8; Ctrl, control; HG/PA, high glucose/palmitic acid; HUVECs, HUVECs, Human umbilical vein endothelial cells; NC, negative control; p-JNK, phosphorylation of JNK; siRNA, small interfering RNA

Now that we have established that the down-regulation of WWP2 aggravates T2DM-induced vascular endothelial injury, our next objective in the current study was to address two aspects: first, to elucidate how endothelial WWP2 expression is down-regulated in during T2DM, and second, to explore how WWP2 regulates T2DM-induced vascular endothelial injury. Studies have shown that HG/PA can increase JNK activation in β-cell which contribute to HG/PA-induced apoptosis [42, 43]. In addition, JNK signaling has been reported to negatively control *WWP2* gene and intronic miR-140-5p via DNMT3α [44]. Hence, we hypothesized that HG/PA can down-regulate the expression of WWP2 in ECs through JNK activation. Western blotting revealed that HG/PA treatment significantly induced activation of JNK in HUVECs, as demonstrated by increased phosphorylation of JNK (p-JNK) (Fig. 5A). To confirm the effect of JNK activation on WWP2 expression levels, we treated HUVECs with JNK inhibitor SP600125 and JNK activator Anisomycin, with or without HG/PA, and assessed WWP2 mRNA and protein levels by real-time PCR and western blotting. We found that HG/PA treatment significantly reduced WWP2 mRNA and protein levels (Fig. 5B–D). Moreover, inhibition of JNK by SP600125 effectively restored WWP2 mRNA and protein levels, while activation of JNK by Anisomycin further decreased WWP2 mRNA and protein levels (Fig. 5B–D), suggesting that HG/PA down-regulates WWP2 through JNK activation. To further confirm that JNK activation inhibited WWP2 expression in HG/PA-induced endothelial injury, HUVECs were transfected with WWP2 siRNA and treated with JNK inhibitor SP600125, with or without HG/PA treatment. As shown in Fig. 5E, inhibition of JNK by SP600125 restored cell proliferation and viability of HUVECs suppressed by HG/PA, but knockdown of WWP2 reversed this tendency. Flow cytometry analysis revealed that inhibition of JNK by SP600125 alleviated HG/PA-induced HUVECs apoptosis, while WWP2 knockdown reversed this effect (Fig. 5F, G). Furthermore, inhibition of JNK by SP600125 decreased the protein levels of apoptosis marker cleaved PARP1 and cleaved caspase-3, which can be reversed by WWP2 knockdown (Fig. 5H, I). Together, these results demonstrate that WWP2 is down-regulated in HG/PA-induced HUVECs due to JNK activation.

### WWP2 interacts with DDX3X

**Fig. 6:**
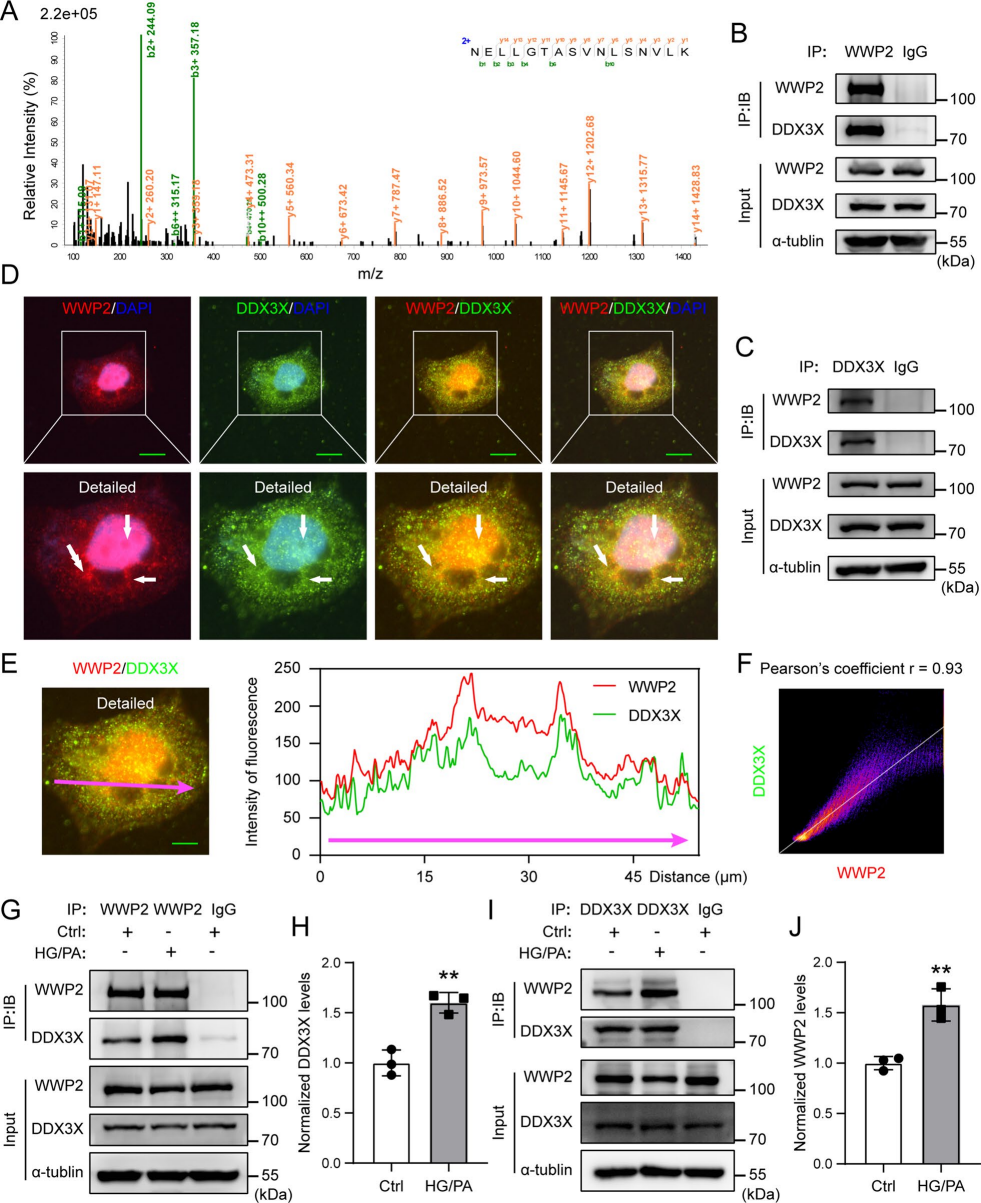
DDX3X was identified to interact with WWP2. **A** Interaction between purified HA-WWP2 and DDX3X analyzed by mass spectroscopy. **B, C** Co-immunoprecipitation analysis of the endogenous interaction between WWP2 and DDX3X. **D** Subcellular localization of WWP2 and DDX3X. HUVECs were stained with WWP2 (red), DDX3X (green) and DAPI (blue). Scale bar, 20 μm. **E** Plot profiles (right panel) showed the fluorescence intensity of WWP2 (red) and DDX3X (green) along the pink indicator line in the merge image (left panel). Scale bar, 10 μm. **F** Scatter plots depicted the distribution of pixels in the WWP2 (red) and DDX3X (green) channels. Pearson’s coefficient r = 0.93. **G–J** HUVECs were stimulated with HG/PA for 48 h, followed by co-immunoprecipitation analysis (**G, I**) and quantitative analysis (**H, J**) of endogenous interactions between WWP2 and DDX3X. Data were quantified from three independent experiments. Values are shown as mean ± SD (**P < 0.01, two-tailed unpaired Student t-tests). DAPI, 4′,6-diamidino-2-phenylindole; HG/PA, high glucose/palmitic acid; HUVECs, Human umbilical vein endothelial cells

In an effort to decipher the potential mechanisms by which WWP2 regulates T2DM-induced vascular endothelial injury, we carried out mass spectrometry to identify the proteins associated with WWP2. Among the copurified proteins, DDX3X was a new and abundant WWP2 binding partner (55 peptides, 41.44% coverage) (Fig. 6A). DDX3X (DEAD-box helicase 3 X-linked) is a member of the large DEAD-box helicase family [45]. It has been reported that the interaction between DDX3X and hnRNPK could play a pro-apoptotic role in U2OS osteosarcoma cells under DNA-damage conditions [46, 47]. In addition, DDX3X serves as a key checkpoint in apoptotic signaling in DNA damage-induced breast cancer cells [48]. Thus, we hypothesized that the interaction between WWP2 and DDX3X play a pivotal role in T2DM-induced vascular endothelial cell injury. To test whether WWP2 interacted with DDX3X, we performed endogenous co-immunoprecipitation assays to verify the interaction between WWP2 and DDX3X in HUVECs (Fig. 6B, C). Next, we explored the spatial relationship between endogenous WWP2 and DDX3X, and found extensive colocalization between WWP2 (red) and DDX3X (green) (Fig. 6D, E). The Pearson’s coefficient of colocalization between WWP2 (red) and DDX3X (green) was 0.93 (Fig. 6F). Furthermore, endogenous co-immunoprecipitation results showed that HG/PA treatment significantly increased the binding between WWP2 and DDX3X in HUVECs (Fig. 6G–J). These results demonstrate that WWP2 interacts with DDX3X and that WWP2 may be involved in endothelial injury through its interaction with DDX3X.

### WWP2 E3 ligase reduces the protein level of DDX3X by promoting its K63-linked polyubiquitination and proteasomal degradation

**Fig. 7:**
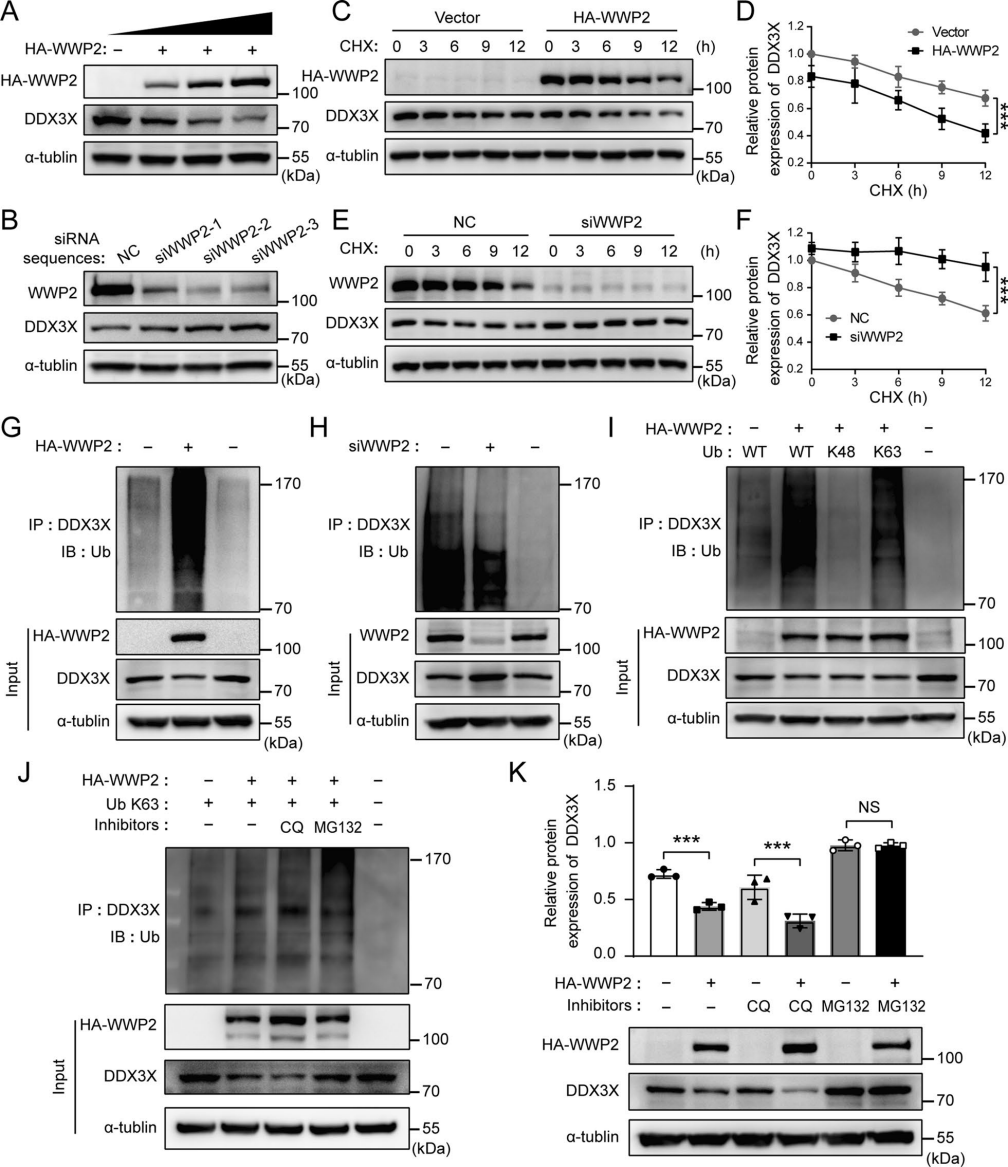
WWP2 reduces the protein level of DDX3X by promoting its K63-linked polyubiquitination and proteasomal degradation. **A, B** Representative Western blots of WWP2 and DDX3X protein levels. HUVECs were transfected with expression plasmids encoding HA-WWP2 (**A**) or three siRNA sequences of WWP2 (**B**). **C–F** Representative Western blotting analysis of WWP2 and DDX3X protein levels (**C, E**) and quantitative analysis of DDX3X protein expression levels (**D, F**). HUVECs were transfected with expression plasmid encoding HA-WWP2 (**C, D**) or WWP2 siRNA (**E, F**) and then treated with CHX (20 μM) for the indicated time periods. Values are shown as mean ± SD (***P < 0.001, two-way ANOVA with Bonferroni’s multiple comparison post hoc test). **G, H** Co-immunoprecipitation analysis was performed to assess the role of WWP2 in regulating the levels of DDX3X ubiquitination. HUVECs were transfected with HA-WWP2 plasmid (**G**) or WWP2 siRNA (**H**), and the levels of DDX3X ubiquitylation were analyzed by immunoprecipitation of DDX3X followed by anti-HA or anti-Ub immunoblotting. **I** Effects of the indicated K-only ubiquitin mutants (K48 and K63) on WWP2-mediated DDX3X polyubiquitination. HUVECs were transfected with the indicated constructs. **J** Effects of lysosome inhibitor CQ and proteasome inhibitor MG132 on WWP2-mediated K63-linked polyubiquitination of DDX3X. HUVECs were transfected with expression plasmids encoding HA-WWP2 and Ub K63, and then treated with 50 μM CQ for 24 h or 10 μM MG132 for 6 h, or DMSO as control. **K, L** Representative Western blotting analysis of HA-WWP2 and DDX3X protein levels (**K**) and quantitative analysis of DDX3X protein expression levels (**L**). Values are shown as mean ± SD (***P < 0.001, NS = not significant, two-way ANOVA with Bonferroni’s multiple comparison post hoc test). CHX, cycloheximide; CQ, chloroquine; HUVECs, Human umbilical vein endothelial cells; NC, negative control; siRNA, small interfering RNA; Ub, ubiquitin

Given that WWP2 is a HECT domain–containing E3 Ub ligase, we next investigated whether it regulates DDX3X ubiquitination and stability. Overexpression of WWP2 dose-dependently decreased the protein level of DDX3X (Fig. 7A), while knockdown of WWP2 by three WWP2 siRNA sequences obviously increased the protein expression of DDX3X (Fig. 7B). To investigate whether the decrease in DDX3X protein levels in the presence of WWP2 was due to an increase in DDX3X degradation, we analyzed DDX3X turnover by treating HUVECs with CHX, an inhibitor of protein synthesis. The results showed that overexpression of WWP2 significantly shortened the half-life of DDX3X protein (Fig. 7C, D), whereas knockdown of WWP2 had the opposite effect in HUVECs (Fig. 7E, F), demonstrating that WWP2 promotes degradation of the DDX3X protein.

Protein ubiquitination is a multifaceted post-translational modification involved in various pathological processes such as vascular endothelial injury [49, 50]. Ubiquitin (Ub) can be ubiquitinated at its seven Lys residues (K6, K11, K27, K29, K33, K48, and K63) to monoubiquitylate or polyubiquitinate target substrates by specific E3 Ub ligases [51]. Among the seven chain types, K48-linked ubiquitin chains are the predominant linkage type in cells that targets proteins for proteasomal degradation [52]. Similarly, the second most abundant Ub chain type linked through K63 can also perform proteolytic functions in a proteasome- or lysosome- dependent manner [53, 54]. To explore whether E3 Ub ligase WWP2 mediates the DDX3X ubiquitination, we assessed the levels of DDX3X ubiquitylation in HUVECs with overexpressed or silenced WWP2 by co-immunoprecipitation assay. Overexpression of WWP2 markedly increased the levels of DDX3X polyubiquitylation (Fig. 7G), whereas knockdown of WWP2 using a siRNA reduced the levels of DDX3X polyubiquitylation (Fig. 7H), revealing that WWP2 mediates DDX3X polyubiquitination.

Since K48-linked and K63-linked Ub chains are the most dominant chain types in cells, we next focused on whether DDX3X underwent these two Ub chain types generated by WWP2. As shown in Fig. 7I, K63-only Ub (with K63 lysine residue retained and the other 6 lysine residues mutated to arginine), but not K48-only Ub, enabled the polyubiquitination of DDX3X similar to WT Ub. The finding suggests that the WWP2-catalyzed poly-Ub modification of DDX3X is primarily dependent on the presence of the K63-linkage. Furthermore, WWP2-mediated K63-linked polyubiquitination of DDX3X was significantly enhanced by proteasome inhibitor MG132 but not lysosome inhibitor CQ (Fig. 7J). Consistently, WWP2-mediated degradation of DDX3X was considerably hindered by MG132 rather than CQ (Fig. 7K), suggesting WWP2-induced degradation of DDX3X through proteasome pathway.

K63-linked Ub can perform proteolytic functions in a proteasome- or lysosome- dependent manner [53, 54]. However, CQ showed no effects on WWP2-mediated K63-polyubiquitination and degradation of DDX3X (Fig. 7J, K). Is it possibly because that treatment with 50 µM CQ failed to inhibit lysosomal degradation in HUVECs. To determine whether treatment with 50 µM CQ inhibited lysosomal degradation in HUVECs, HUVECs were transfected with expression plasmids encoding HA-WWP2, with or without Ub K63, and then treated with 0, 20, 50, 100 μM CQ for 24 h. Previous research has reported that WWP2 can be degraded through the lysosome pathway [55]. As shown in Additional file 1: Fig. S2A–C, our results showed that treatment with 20, 50, and 100 μM CQ significantly up-regulated the expression of HA-WWP2, suggesting that 50 μM CQ could successfully inhibit lysosomal degradation in HUVECs. However, treatment with CQ showed no effects on WWP2-mediated K63-polyubiquitination and degradation of DDX3X (Additional file 1: Fig. S2A–C), further confirming our conclusion that WWP2-mediated Ub-dependent degradation of DDX3X is via the proteasome pathway rather than the lysosome pathway.

Next, we confirmed the regulation of DDX3X by WWP2 in vivo. Due to the complex cell types in vascular tissues and the small proportion of endothelial cells, it is difficult to get sufficient endothelial cells from mouse vascular tissue for co-immunoprecipitation and ubiquitination tests. Therefore, to eliminate interference from other cell types, we adopted a more precise co-immunofluorescent staining assays to establish the impact of endothelium-specific *Wwp2* knockout on endothelial DDX3X expression. Compared with Cdh5 Cre-; *Wwp2*^*fl/fl*^ mice, endothelial-specific *Wwp2* knockout mice (Cdh5 Cre + ; *Wwp2*^*fl/fl*^ mice) showed elevated endothelial DDX3X expression under both normal conditions (Additional file 1: Fig. S3A, B) and T2DM conditions (Additional file 1: Fig. S3C, D), confirming that WWP2 reduces the protein level of DDX3X in vivo. Together, our results demonstrated that WWP2 reduces the protein level of DDX3X by promoting its K63-linked polyubiquitination and proteasomal degradation.

### DDX3X mediates the regulation of WWP2 on endothelial injury

**Fig. 8:**
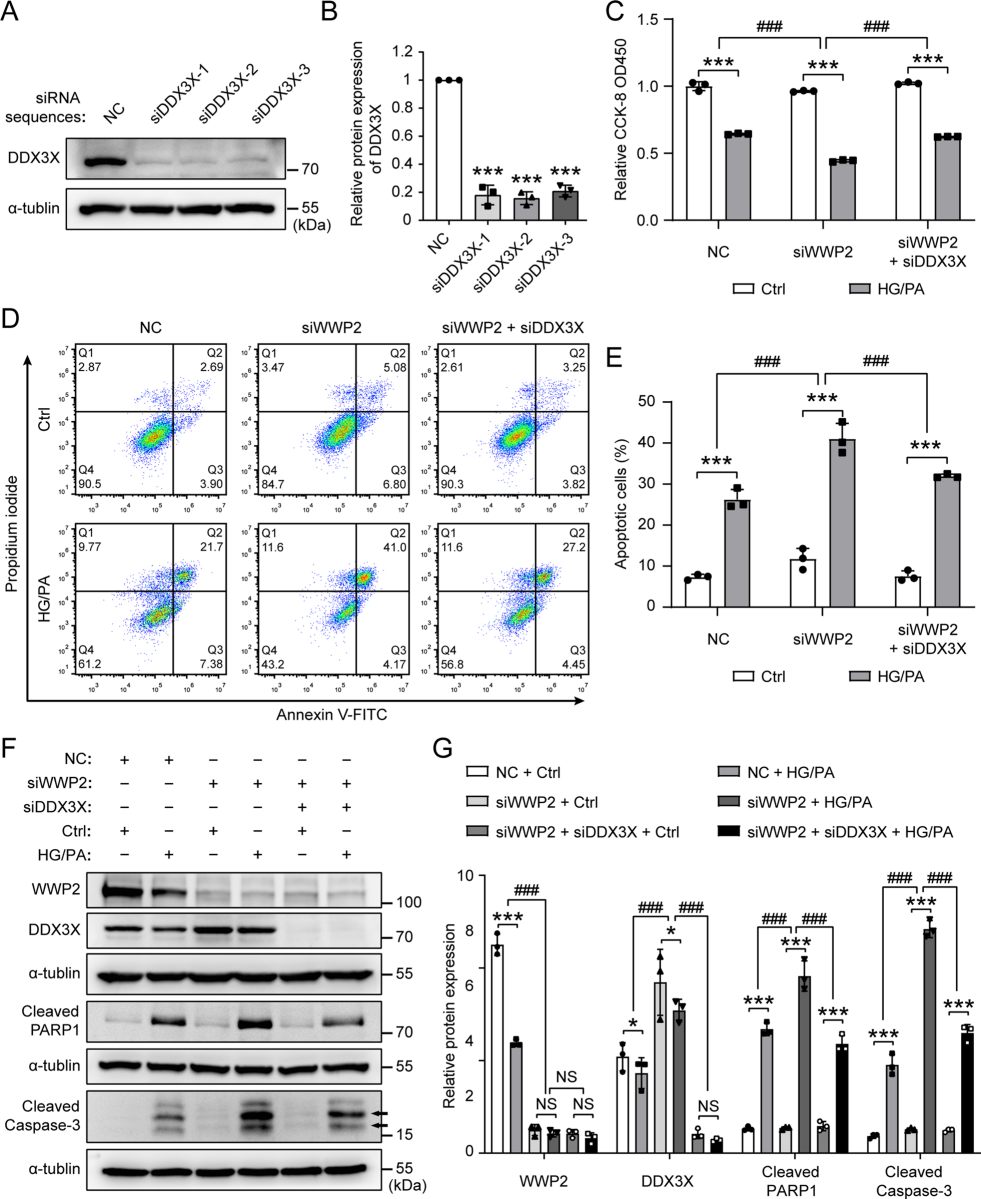
DDX3X is essential for the regulation of WWP2 on endothelial injury. **A**, **B** Representative Western blots (**A**) and quantification (**B**) of DDX3X protein expression levels in HUVECs. HUVECs were transfected with three siRNA sequences of DDX3X to evaluate the efficiency of DDX3X silencing. Values are shown as mean ± SD (***P < 0.001, one-way ANOVA with Dunnett’s multiple comparison post-hoc test). **c** CCK-8 colorimetric assay was used to test the proliferation and viability of HUVECs in each indicated group. HUVECs were transfected with DDX3X siRNA or a negative control, and transfected with WWP2 siRNA or a negative control, with or without HG/PA treatment. **D**, **E** Representative Flow cytometry analysis (**D**) and quantitative analysis (**E**) of HUVECs apoptosis in each indicated group. **F**, **G** Representative Western blots (**F**) and quantification (**G**) of WWP2, DDX3X, Cleaved PARP1 and Cleaved caspase-3 protein expression levels in HUVECs of each indicated group. Values are shown as mean ± SD (*P < 0.05, ***P < 0.001, ###P < 0.001, NS = not significant, two-way ANOVA with Bonferroni’s multiple comparison post hoc test). CCK-8, Cell Counting Kit-8; Ctrl, control; HG/PA, high glucose/palmitic acid; HUVECs, HUVECs, Human umbilical vein endothelial cells; NC, negative control; siRNA, small interfering RNA

**Fig. 9:**
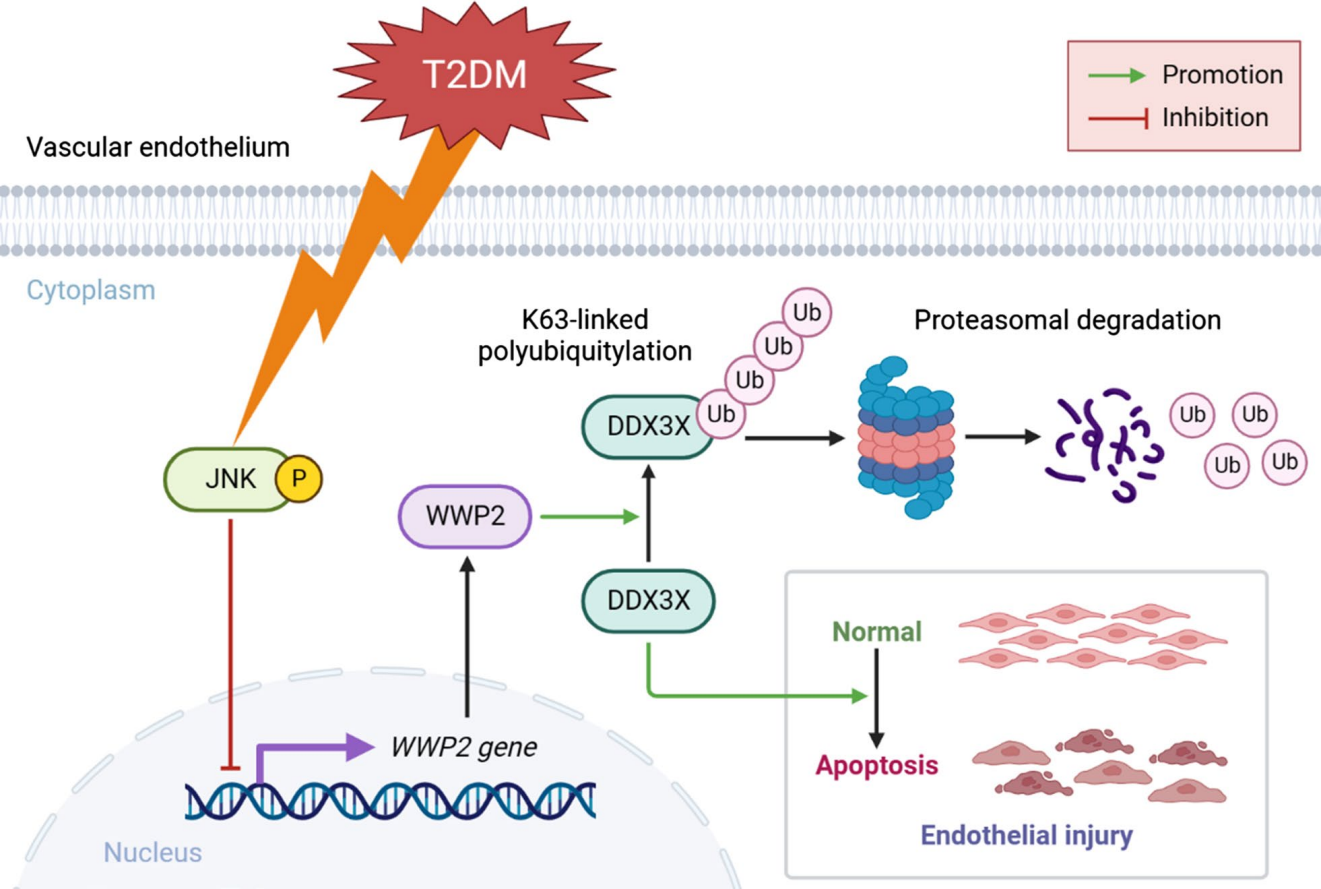
Schematic illustration of WWP2 function in T2DM-induced vascular endothelial injury. Down-regulation of WWP2 triggered by JNK activation exacerbates T2DM-induced vascular endothelial injury through modulating K63-linked polyubiquitination and proteasomal degradation of DDX3X. p-JNK, phosphorylation of JNK; T2DM, Type 2 diabetes mellitus

To further verify the role of DDX3X in the regulation of endothelial injury by WWP2, we engineered three siRNAs to knock down DDX3X in HUVECs. The efficiency of DDX3X silencing was assessed by western blotting and the most effective siRNA sequence was used for subsequent experiments (Fig. 8A, B). As shown in Fig. 8C, knockdown of DDX3X using a siRNA reversed the inhibitory effect of WWP2 knockdown on HUVECs survival. Flow cytometry analysis revealed that WWP2 knockdown-induced apoptosis was significantly rescued in DDX3X knockdown HUVECs (Fig. 8D, E). Moreover, DDX3X knockdown in HUVECs reversed the effect of WWP2 knockdown on levels of cleaved PARP1 and cleaved caspase-3 (Fig. 8F, G). These findings demonstrated that DDX3X mediates the regulation of WWP2 on endothelial injury. Taken as a whole, our results revealed that down-regulation of WWP2 triggered by JNK activation exacerbates T2DM-induced vascular endothelial injury through modulating K63-linked polyubiquitination and proteasomal degradation of DDX3X (Fig. 9). Taken together, the present study revealed the crucial function of WWP2 and the fundamental importance of the JNK-WWP2-DDX3X regulatory axis in T2DM-induced vascular endothelial injury, suggesting that JNK-WWP2-DDX3X axis has potential as a preventive and therapeutic target for DVCs.

## Discussion

Endothelial injury caused by chronic T2DM is considered as a mainstay in the pathophysiology of DVCs [6–8]. In the present study, we identified E3 Ub ligase WWP2 as a novel regulator in T2DM-induced vascular endothelial injury by analyzing single-cell transcriptome profiles of ECs from T2DM donors and evaluating the expression of WWP2 in in vivo and in vitro experiments. Endothelial-specific *Wwp2* knockout in mice significantly aggravated T2DM-induced vascular endothelial injury and vascular remodeling after endothelial injury. Similarly, our in vitro experiments showed that WWP2 prevents endothelial injury by maintaining cell survival and inhibiting apoptosis. Mechanically, we found that WWP2 is down-regulated in HG/PA-induced HUVECs due to JNK activation. Furthermore, we identified DDX3X as a new interaction partner of WWP2, and discovered that WWP2 protects against T2DM-induced endothelial injury by targeting DDX3X for K63-linked polyubiquitination and proteasomal degradation (Fig. 9). Therefore, WWP2 and is important in the pathogenesis of T2DM-induced vascular endothelial injury.

Protein ubiquitination is a critical post-translational modification that affects multiple diseases processes, including vascular endothelial injury [49, 50]. Once attached to the substrate protein, Ub can be further modified by specific E3 Ub ligases at its lysine residues to form polyubiquitin chains encompassing complex topologies that create a wide diversity of distinct biological signals with distinct cellular outcomes [51]. K48-linked Ub chains are the most predominant linkage type in cells that primarily targets substrate proteins for proteasomal degradation [52]. Similarly, the second most abundant Ub chain type linked through K63 can also perform proteolytic functions in a proteasome- or lysosome- dependent manner [53, 54]. Emerging evidence showed that K63‐linked ubiquitination plays a key role in the regulation of endothelial barrier function and endothelial inflammatory response [56, 57]. Nevertheless, the physiological role of K63-linked polyubiquitination is unknown in T2DM-induced vascular endothelial injury. In this study, we indicated that E3 Ub ligase WWP2 can degrade DDX3X by catalyzing its K63-linked polyubiquitination to regulate T2DM-induced vascular endothelial injury. Our results provide an example of K63-linked polyubiquitination regulating substrate protein stability and T2DM-induced vascular endothelial injury.

WWP2 is an E3 Ub ligase that regulates a variety of physiological and pathological processes by regulating ubiquitination and degradation of specific substrates. WWP2 physically interacts with the tumor suppressor PTEN and promotes proteasome-mediated degradation of PTEN to induce tumorigenesis [14]. WWP2 also promotes degradation of transcription factor OCT4, which plays a fundamental role in maintaining the self-renewing and pluripotent state of embryonic stem cells [10]. Moreover, WWP2 participates in the regulation of immune response and cartilage homeostasis by catalyzing TLR3 ubiquitination [12] and RUNX2 ubiquitination [11], respectively. Furthermore, WWP2-mediated degradation mechanism of sphingosine-1-phosphate receptor 1 is critical for vascular leak in vivo [30]. However, it is not yet clear whether WWP2 is associated with T2DM-induced vascular endothelial injury. Of note, our present study observed that WWP2 is involved in T2DM-induced vascular endothelial injury by single-cell analysis, in vivo and in vitro experiments. Recently, Zhang et al. using Tek-Cre system for endothelial/myeloid-specific WWP2 deletion showed that WWP2 modulating angiotensinII-induced oxidative stress in endothelial cells by inducing the ubiquitination and proteasome-mediated degradation of septin4 [21]. However, the Tek-Cre system is nonspecific and may target several immune cell types that have a dramatic impact on angiotensinII-induced endothelial cell functions [58, 59]. Hence, we adopted the Cdh5-cre system for endothelial-specific *Wwp2* knockout to avoid this effect and found that endothelial-specific loss of *Wwp2* in mice significantly aggravated T2DM-induced vascular endothelial injury and vascular remodeling after endothelial injury. In addition, in vitro experiments suggested that WWP2 protects against endothelial injury by maintaining cell survival and inhibiting apoptosis. Our results identify a novel and critical protective role of WWP2 in the process of T2DM-induced vascular endothelial injury.

Now that we have established that the down-regulation of WWP2 exacerbates T2DM-induced vascular endothelial injury, we then addressed two aspects: first, to elucidate how endothelial WWP2 expression is down-regulated in during T2DM, and second, to explore how WWP2 regulates T2DM-induced vascular endothelial injury. Previous researches have reported HG/PA can increase JNK activation, which is known to be a key mediator of HG/PA-induced apoptosis [42, 43]. In addition, JNK signaling has been reported to negatively control *WWP2* gene and intronic miR-140-5p via DNMT3α [44]. Consistently, the current study found that HG/PA down-regulates WWP2 by inducing JNK activation, which contributes to HG/PA-induced endothelial injury.

Next, we deciphered the potential mechanisms by which WWP2 regulates T2DM-induced vascular endothelial injury. DDX3X is a member of the DEAD-box helicase family and is engaged in various cellular processes, including cell cycle progression, cellular differentiation, cell survival, and apoptosis [45, 60]. The interaction between DDX3X and hnRNPK has been reported in the context of the apoptosis of pancreatic β cells [46]. Moreover, the interaction between DDX3X and hnRNPK could play a pro-apoptotic role in U2OS osteosarcoma cells under DNA-damage conditions [47]. In breast cancer cells, DDX3X serves as a key checkpoint in apoptotic signaling [48]. Specifically, DDX3X positively regulates apoptotic signaling in cells expressing functional wild-type p53, whereas DDX3X inhibits activation of apoptotic signaling in cells expressing mutant or nonfunctional p53 [48]. In spite of these studies indicated that DDX3X is an important regulator of tumor cell apoptosis, the functional role of DDX3X in endothelial injury was unclear. Here, we found that DDX3X is a new interaction partner of WWP2 and is essential for the protective effect of WWP2 on T2DM-induced vascular endothelial injury. DDX3X can be regulated by the Ub system via binding to and ubiquitination by a variety of different E3 Ub ligases. The E3 ligase TRIM25 increases K63-linked ubiquitination of DDX3X to controlling antiviral immunity [61]. RING finger protein 39 (RNF39) suppresses retinoic acid–inducible gene-I (RIG-I)-like receptors (RLR)-dependent antiviral immunity by promoting K48-linked polyubiquitination and proteasomal degradation of DDX3X [62]. In the present study, we demonstrated that E3 Ub ligase WWP2 decorates DDX3X with K63-linked polyubiquitin chains and targets it for proteasomal degradation, thereby regulating T2DM-induced vascular endothelial injury. Collectively, our data provide new insights into the involvement of DDX3X in endothelial injury and the regulation of DDX3X protein stability.

Our study also has several limitations. First, while we used only male samples in order to avoid potential variations contributed by gender and genetic background, the overrepresentation of male tissues failed to assess the effect of sex differences on molecular mechanisms underlying T2DM-induced vascular endothelial injury. So, our future work will 1increase the sample size to include both male and female samples, and implement a sex-stratified approach to observe sex differences on molecular mechanisms underlying T2DM-induced vascular endothelial injury. Second, although we have demonstrated the protective effect of WWP2 on T2DM-induced vascular endothelial injury through in vivo and in vitro experiments, the function of WWP2 in patients with T2DM warrants further investigation. Third, while we described here a novel role of WWP2 in T2DM-induced vascular endothelial injury and vascular remodeling after endothelial injury, more research is needed to clarify the role of WWP2-regulated T2DM-induced vascular endothelial injury in DVCs.

In summary, we discovered that WWP2 is down-regulated in HG/PA-induced ECs due to JNK activation, and that WWP2 regulates HG/PA-induced endothelial injury by targeting DDX3X for K63-linked polyubiquitination and proteasomal degradation. Our findings uncover that WWP2 is a novel regulator of T2DM-induced vascular endothelial injury, and that the JNK/WWP2/DDX3X axis is associated with HG/PA induced endothelial injury, providing a new molecular basis for the treatment and prevention of DVCs.

## Conclusions

In conclusion, in this study, we unveiled that down-regulation of WWP2 triggered by JNK activation exacerbates aggravates T2DM-induced vascular endothelial injury through modulating K63-linked polyubiquitination and proteasomal degradation of DDX3X. The present study revealed the key role of endothelial WWP2 and the fundamental importance of the JNK-WWP2-DDX3X regulatory axis in T2DM-induced vascular endothelial injury, suggesting that WWP2 may serve as a new therapeutic target for DVCs.

## Supplementary Information

**Additional file 1: Fig. S1** Single-cell analysis of endothelial cells from healthy and T2DM donors. A, B UMAP plot showing 6,917 mesenteric arterial cells isolated from 2 healthy control (N) and 1 T2DM (T) male donors. Number of cells sequenced are: N1_1: 1069, N1_2: 729, N2_1: 389, N2_2: 382, N2_3: 406, N2_4: 306, T2_1: 1814, T2_2: 1822. C UMAP plot showing clusters identified by graph-based semi-unsupervised clustering. D Dot plot of top five traditional markers used for cell identity. E, F Feature plots showing gene expression of Cdh5 (E) and Pecam1 (F), as representative genes of classical markers for endothelial cells. Ctrl, control; T2DM, Type 2 diabetes mellitus; UMAP, Uniform Manifold Approximation and Projection. **Fig. S2** WWP2-mediated Ubiquitin (Ub)-dependent degradation of DDX3X is not a lysosome pathway. A Effects of lysosome inhibitor chloroquine (CQ) on WWP2-mediated K63-linked polyubiquitination of DDX3X. Human umbilical vein endothelial cells (HUVECs) were transfected with expression plasmids encoding HA-WWP2 and Ub K63, and then treated with the indicated concentrations of CQ for 24 h or DMSO as control. B, C Representative Western blotting analysis (B) and quantitative analysis (C) of HA-WWP2 and DDX3X protein levels. HUVECs were transfected with expression plasmids encoding HA-WWP2, and then treated with the indicated concentrations of CQ for 24 h or DMSO as control. Values are shown as mean ± SD (***P < 0.001, one-way ANOVA with Dunnett’s multiple comparison post-hoc test). **Fig. S3** Endothelial-specific Wwp2 knockout in mice leads to up-regulation of endothelial DDX3X expression. A, B, C, D Representative immunofluorescent staining of aortic cross sections for the indicated mice (A, C) and quantitative analysis of relative fluorescence intensity of DDX3X in CD31 positive cells (B, D). (n = 6 mice per group). Red, DDX3X; Green, CD31 (a marker of ECs); blue, DAPI. scale bar 50 µm. (***P < 0.001, two-tailed unpaired Student t-tests). Cdh5 Cre-, (Cdh5 Cre-; Wwp2fl/fl mice); Cdh5 Cre+, (Cdh5 Cre+; Wwp2fl/fl mice); DAPI, 4′,6-diamidino-2-phenylindole; ECs, Endothelial cells; T2DM, Type 2 diabetes mellitus. **Table S1**. The clinical information of donors in the GSE156341 dataset. BMI, Body Mass Index; HC, healthy control; T2DM, Type 2 diabetes mellitus. **Table S2**. Cell types_top50_markers. Cell types were identified according to the expression of top 50 known markers. **Table S3**. Expression changes of genes in KEGG apoptosis pathway. NS, not significant.

## Data Availability

The datasets generated and/or analysed during the current study are available in the Gene Expression Omnibus (GEO, http://www.ncbi.nlm.nih.gov/geo/), reference number GSE156341. All data generated or analysed during this study are included in this published article (and its Additional files).

## Abbreviations

T2DM: Type 2 diabetes mellitus
DVCs: Diabetic vascular complications
WWP2: WW domain-containing E3 ubiquitin protein ligase 2
AIP2: Atrophin-1 interacting protein 2
Ub: Ubiquitin
ECs: Endothelial cells
HG/PA: High glucose and palmitic acid
JNK: C-Jun N-terminal kinase
DDX3X: DEAD-box helicase 3 X-linked
CCA: Canonical correlation analysis
PCs: Principal components
UMAP: Uniform Manifold Approximation and Projection
HFD: High-fat diet
STZ: Streptozotocin
ND: Normal diet
TG: Triglyceride
TC: Total cholesterol
RT: Room temperature
DAPI: 4′,6-Diamidino-2-phenylindole
HUVECs: Human umbilical vein endothelial cells
H&E: Hematoxylin and eosin
ECGs: Endothelial cell growth supplement
FBS: Fetal bovine serum
MAN: Mannitol
CHX: Cycloheximide
CQ: Chloroquine
PCR: Polymerase chain reaction
Ct: Cycle thresholds
siRNAs: Small interfering RNAs
CCK-8: Cell Counting Kit-8
NS: Not significant
p-JNK: Phosphorylation of JNK

## References

[1] Magliano DJ (2021). Trends in the incidence of diagnosed diabetes: a multicountry analysis of aggregate data from 22 million diagnoses in high-income and middle-income settings. Lancet Diabetes Endocrinol.

[2] Cole JB, Florez JC (2020). Genetics of diabetes mellitus and diabetes complications. Nat Rev Nephrol.

[3] Zheng Y, Ley SH, Hu FB (2018). Global aetiology and epidemiology of type 2 diabetes mellitus and its complications. Nat Rev Endocrinol.

[4] Nauck MA, Wefers J, Meier JJ (2021). Treatment of type 2 diabetes: challenges, hopes, and anticipated successes. Lancet Diabetes Endocrinol.

[5] Wasserman DH, Wang TJ, Brown NJ (2018). The vasculature in prediabetes. Circ Res.

[6] Zhou Z (2018). Erythrocytes from patients with type 2 diabetes induce endothelial dysfunction via arginase I. J Am Coll Cardiol.

[7] Chen S (2021). Impact of glycemic control on the association of endothelial dysfunction and coronary artery disease in patients with type 2 diabetes mellitus. Cardiovasc Diabetol.

[8] Xu J, Zou MH (2009). Molecular insights and therapeutic targets for diabetic endothelial dysfunction. Circulation.

[9] Martin-Serrano J (2005). HECT ubiquitin ligases link viral and cellular PPXY motifs to the vacuolar protein-sorting pathway. J Cell Biol.

[10] Xu H (2009). WWP2 promotes degradation of transcription factor OCT4 in human embryonic stem cells. Cell Res.

[11] Mokuda S (2019). Wwp2 maintains cartilage homeostasis through regulation of Adamts5. Nat Commun.

[12] Yang Y (2013). E3 ligase WWP2 negatively regulates TLR3-mediated innate immune response by targeting TRIF for ubiquitination and degradation. Proc Natl Acad Sci USA.

[13] Aki D (2018). The E3 ligases Itch and WWP2 cooperate to limit TH2 differentiation by enhancing signaling through the TCR. Nat Immunol.

[14] Maddika S (2011). WWP2 is an E3 ubiquitin ligase for PTEN. Nat Cell Biol.

[15] Mahlokozera T (2021). Competitive binding of E3 ligases TRIM26 and WWP2 controls SOX2 in glioblastoma. Nat Commun.

[16] Shao R (2016). Cdh1 regulates craniofacial development via APC-dependent ubiquitination and activation of Goosecoid. Cell Res.

[17] Zhang N (2021). Deacetylation-dependent regulation of PARP1 by SIRT2 dictates ubiquitination of PARP1 in oxidative stress-induced vascular injury. Redox Biol.

[18] Chen H (2019). WWP2 regulates pathological cardiac fibrosis by modulating SMAD2 signaling. Nat Commun.

[19] Calandrelli R (2020). Stress-induced RNA-chromatin interactions promote endothelial dysfunction. Nat Commun.

[20] Zhang N (2020). Selective targeting of ubiquitination and degradation of PARP1 by E3 ubiquitin ligase WWP2 regulates isoproterenol-induced cardiac remodeling. Cell Death Differ.

[21] Zhang N (2020). Role of WW domain E3 ubiquitin protein ligase 2 in modulating ubiquitination and degradation of Septin4 in oxidative stress endothelial injury. Redox Biol.

[22] Kautzky-Willer A, Harreiter J, Pacini G (2016). Sex and gender differences in risk, pathophysiology and complications of type 2 diabetes mellitus. Endocr Rev.

[23] de Ritter R (2023). Sex differences in body composition in people with prediabetes and type 2 diabetes as compared with people with normal glucose metabolism: the Maastricht Study. Diabetologia.

[24] Prospective Studies C and Asia Pacific Cohort Studies (2018). Asia Pacific Cohort Studies, Sex-specific relevance of diabetes to occlusive vascular and other mortality: a collaborative meta-analysis of individual data from 980 793 adults from 68 prospective studies. Lancet Diabetes Endocrinol.

[25] Donahue RP (2007). Sex differences in endothelial function markers before conversion to pre-diabetes: does the clock start ticking earlier among women? The Western New York Study. Diabetes Care.

[26] Hao Y (2021). Integrated analysis of multimodal single-cell data. Cell.

[27] Hafemeister C, Satija R (2019). Normalization and variance stabilization of single-cell RNA-seq data using regularized negative binomial regression. Genome Biol.

[28] Soneson C, Robinson MD (2018). Bias, robustness and scalability in single-cell differential expression analysis. Nat Methods.

[29] You S (2021). Comprehensive bioinformatics analysis identifies POLR2I as a key gene in the pathogenesis of hypertensive nephropathy. Front Genet.

[30] Oo ML (2011). Engagement of S1P(1)-degradative mechanisms leads to vascular leak in mice. J Clin Invest.

[31] Coppari R, Bjorbaek C (2012). Leptin revisited: its mechanism of action and potential for treating diabetes. Nat Rev Drug Discov.

[32] Robertson RP (2004). Beta-cell glucose toxicity, lipotoxicity, and chronic oxidative stress in type 2 diabetes. Diabetes.

[33] An X (2016). Mesenchymal stem cells ameliorated glucolipotoxicity in HUVECs through TSG-6. Int J Mol Sci.

[34] Kong AP (2013). Diabetes and its comorbidities—where East meets West. Nat Rev Endocrinol.

[35] Huang HJ (2007). Intrahippocampal administration of A beta(1–40) impairs spatial learning and memory in hyperglycemic mice. Neurobiol Learn Mem.

[36] Cheng Y (2019). Pancreatic kallikrein protects against diabetic retinopathy in KK Cg-A(y)/J and high-fat diet/streptozotocin-induced mouse models of type 2 diabetes. Diabetologia.

[37] Wu L (2021). The Attenuation of Diabetic Nephropathy by Annexin A1 via Regulation of Lipid Metabolism Through the AMPK/PPARalpha/CPT1b Pathway. Diabetes.

[38] Xu T (2018). Vascular endothelial growth factor over-expressed mesenchymal stem cells-conditioned media ameliorate palmitate-induced diabetic endothelial dysfunction through PI-3K/AKT/m-TOR/eNOS and p38/MAPK signaling pathway. Biomed Pharmacother.

[39] Chen MJ (2009). Runx1 is required for the endothelial to haematopoietic cell transition but not thereafter. Nature.

[40] Hill MA (2021). Insulin resistance, cardiovascular stiffening and cardiovascular disease. Metabolism.

[41] Kaur R, Kaur M, Singh J (2018). Endothelial dysfunction and platelet hyperactivity in type 2 diabetes mellitus: molecular insights and therapeutic strategies. Cardiovasc Diabetol.

[42] Han SJ (2012). beta-Cell-protective effect of 2-aminobicyclo-(2,2,1)-heptane-2-carboxylic acid as a glutamate dehydrogenase activator in db/db mice. J Endocrinol.

[43] Lee JH (2014). Toxicity generated through inhibition of pyruvate carboxylase and carnitine palmitoyl transferase-1 is similar to high glucose/palmitate-induced glucolipotoxicity in INS-1 beta cells. Mol Cell Endocrinol.

[44] Duan P (2022). Intronic miR-140-5p contributes to beta-cypermethrin-mediated testosterone decline. Sci Total Environ.

[45] Mo J (2021). DDX3X: structure, physiologic functions and cancer. Mol Cancer.

[46] Good AL (2019). Metabolic stress activates an ERK/hnRNPK/DDX3X pathway in pancreatic beta cells. Mol Metab.

[47] Chen CC (2021). Arginine methylation of hnRNPK inhibits the DDX3-hnRNPK interaction to play an anti-apoptosis role in osteosarcoma cells. Int J Mol Sci.

[48] Sun M (2013). DDX3 regulates DNA damage-induced apoptosis and p53 stabilization. Biochim Biophys Acta.

[49] Lv Y (2018). YAP controls endothelial activation and vascular inflammation through TRAF6. Circ Res.

[50] Stangl K, Stangl V (2010). The ubiquitin-proteasome pathway and endothelial (dys)function. Cardiovasc Res.

[51] Swatek KN, Komander D (2016). Ubiquitin modifications. Cell Res.

[52] Yau R, Rape M (2016). The increasing complexity of the ubiquitin code. Nat Cell Biol.

[53] Liao B, Jin Y (2010). Wwp2 mediates Oct4 ubiquitination and its own auto-ubiquitination in a dosage-dependent manner. Cell Res.

[54] Hou P (2021). A novel selective autophagy receptor, CCDC50, delivers K63 polyubiquitination-activated RIG-I/MDA5 for degradation during viral infection. Cell Res.

[55] Wang Y (2022). LAPTM5 mediates immature B cell apoptosis and B cell tolerance by regulating the WWP2-PTEN-AKT pathway. Proc Natl Acad Sci USA.

[56] Qian Y (2022). TRIM47 is a novel endothelial activation factor that aggravates lipopolysaccharide-induced acute lung injury in mice via K63-linked ubiquitination of TRAF2. Signal Transduct Target Ther.

[57] Kovacevic I (2018). The Cullin-3-Rbx1-KCTD10 complex controls endothelial barrier function via K63 ubiquitination of RhoB. J Cell Biol.

[58] Guzik TJ (2007). Role of the T cell in the genesis of angiotensin II induced hypertension and vascular dysfunction. J Exp Med.

[59] Wenzel P (2011). Lysozyme M-positive monocytes mediate angiotensin II-induced arterial hypertension and vascular dysfunction. Circulation.

[60] Sun M (2008). Identification of an antiapoptotic protein complex at death receptors. Cell Death Differ.

[61] Atkinson SC (2021). TRIM25 and DEAD-Box RNA helicase DDX3X cooperate to regulate RIG-I-mediated antiviral immunity. Int J Mol Sci.

[62] Wang W, et al. RNF39 mediates K48-linked ubiquitination of DDX3X and inhibits RLR-dependent antiviral immunity. Sci Adv. 2021. **7**(10). 10.1126/sciadv.abe5877 PMC7935364 33674311

