# Supplementary material for: Down-regulation of WWP2 aggravates Type 2 diabetes mellitus-induced vascular endothelial injury through modulating ubiquitination and degradation of DDX3X
Source: Cardiovasc Diabetol. 2023 May 6;22:107. doi: 10.1186/s12933-023-01818-3 (PMC10164326; doi:10.1186/s12933-023-01818-3)
Supplement: Supplementary file 1 — Additional file 1: Fig. S1 Single-cell analysis of endothelial cells from healthy and T2DM donors. A, B UMAP plot showing 6,917 mesenteric arterial cells isolated from 2 healthy control (N) and 1 T2DM (T) male donors. Number of cells sequenced are: N1_1: 1069, N1_2: 729, N2_1: 389, N2_2: 382, N2_3: 406, N2_4: 306, T2_1: 1814, T2_2: 1822. C UMAP plot showing clusters identified by graph-based semi-unsupervised clustering. D Dot plot of top five traditional markers used for cell identity. E, F Feature plots showing gene expression of Cdh5 (E) and Pecam1 (F), as representative genes of classical markers for endothelial cells. Ctrl, control; T2DM, Type 2 diabetes mellitus; UMAP, Uniform Manifold Approximation and Projection. Fig. S2 WWP2-mediated Ubiquitin (Ub)-dependent degradation of DDX3X is not a lysosome pathway. A Effects of lysosome inhibitor chloroquine (CQ) on WWP2-mediated K63-linked polyubiquitination of DDX3X. Human umbilical vein endothelial cells (HUVECs) were transfected with expression plasmids encoding HA-WWP2 and Ub K63, and then treated with the indicated concentrations of CQ for 24 h or DMSO as control. B, C Representative Western blotting analysis (B) and quantitative analysis (C) of HA-WWP2 and DDX3X protein levels. HUVECs were transfected with expression plasmids encoding HA-WWP2, and then treated with the indicated concentrations of CQ for 24 h or DMSO as control. Values are shown as mean ± SD (***P < 0.001, one-way ANOVA with Dunnett’s multiple comparison post-hoc test). Fig. S3 Endothelial-specific Wwp2 knockout in mice leads to up-regulation of endothelial DDX3X expression. A, B, C, D Representative immunofluorescent staining of aortic cross sections for the indicated mice (A, C) and quantitative analysis of relative fluorescence intensity of DDX3X in CD31 positive cells (B, D). (n = 6 mice per group). Red, DDX3X; Green, CD31 (a marker of ECs); blue, DAPI. scale bar 50 µm. (***P < 0.001, two-tailed unpaired Student t-tests). Cdh5 Cre-, (Cdh5 C [file 12933_2023_1818_MOESM1_ESM.docx]

**
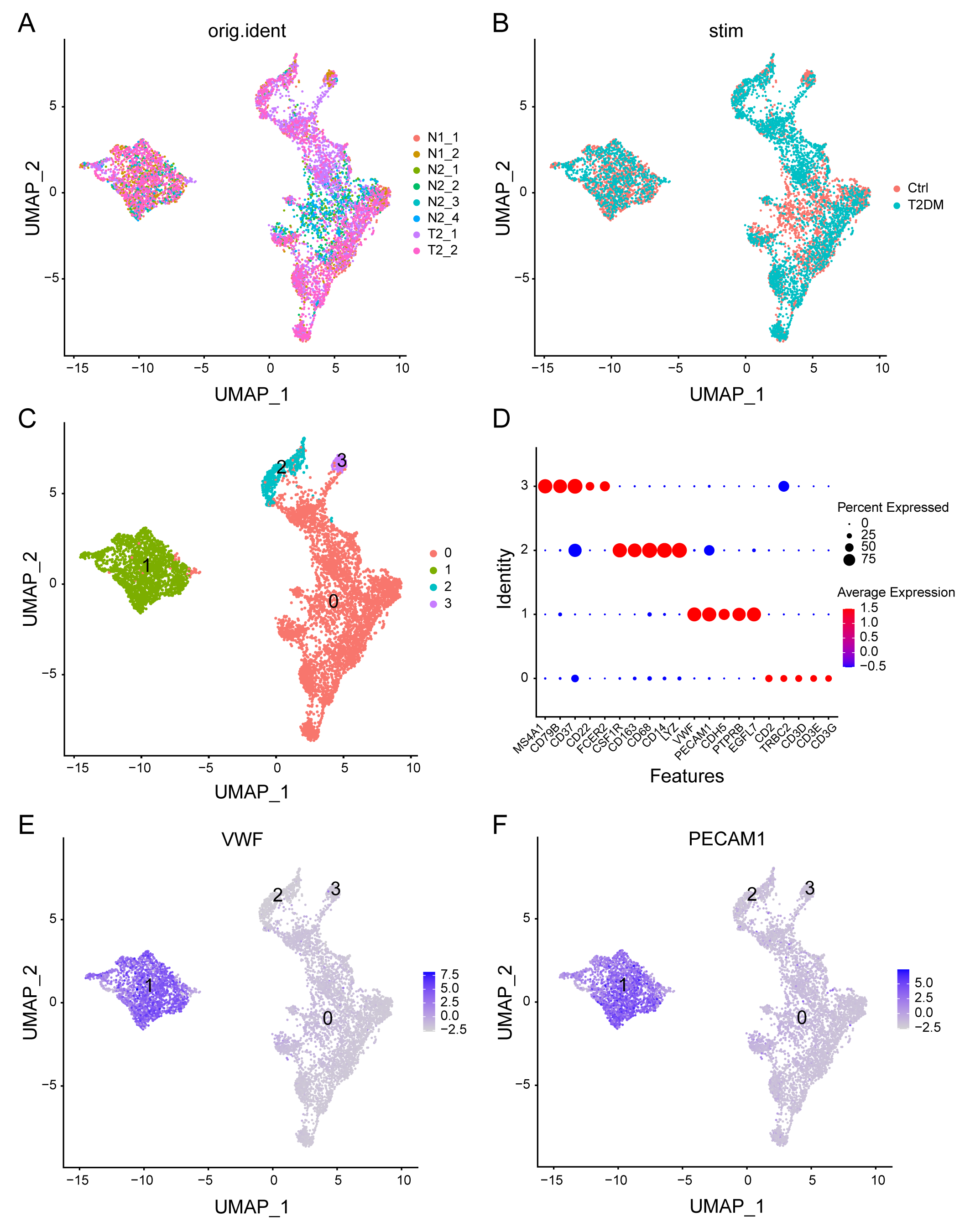
**

**Fig. S1 Single-cell analysis of endothelial cells from healthy and T2DM donors. A, B** UMAP plot showing 6,917 mesenteric arterial cells isolated from 2 healthy control (N) and 1 T2DM (T) male donors. Number of cells sequenced are: N1_1: 1069, N1_2: 729, N2_1: 389, N2_2: 382, N2_3: 406, N2_4: 306, T2_1: 1814, T2_2: 1822. **C** UMAP plot showing clusters identified by graph-based semi-unsupervised clustering. **D** Dot plot of top five traditional markers used for cell identity. **E, F** Feature plots showing gene expression of Cdh5 **(E)** and Pecam1 **(F)**, as representative genes of classical markers for endothelial cells. Ctrl, control; T2DM, Type 2 diabetes mellitus; UMAP, Uniform Manifold Approximation and Projection.


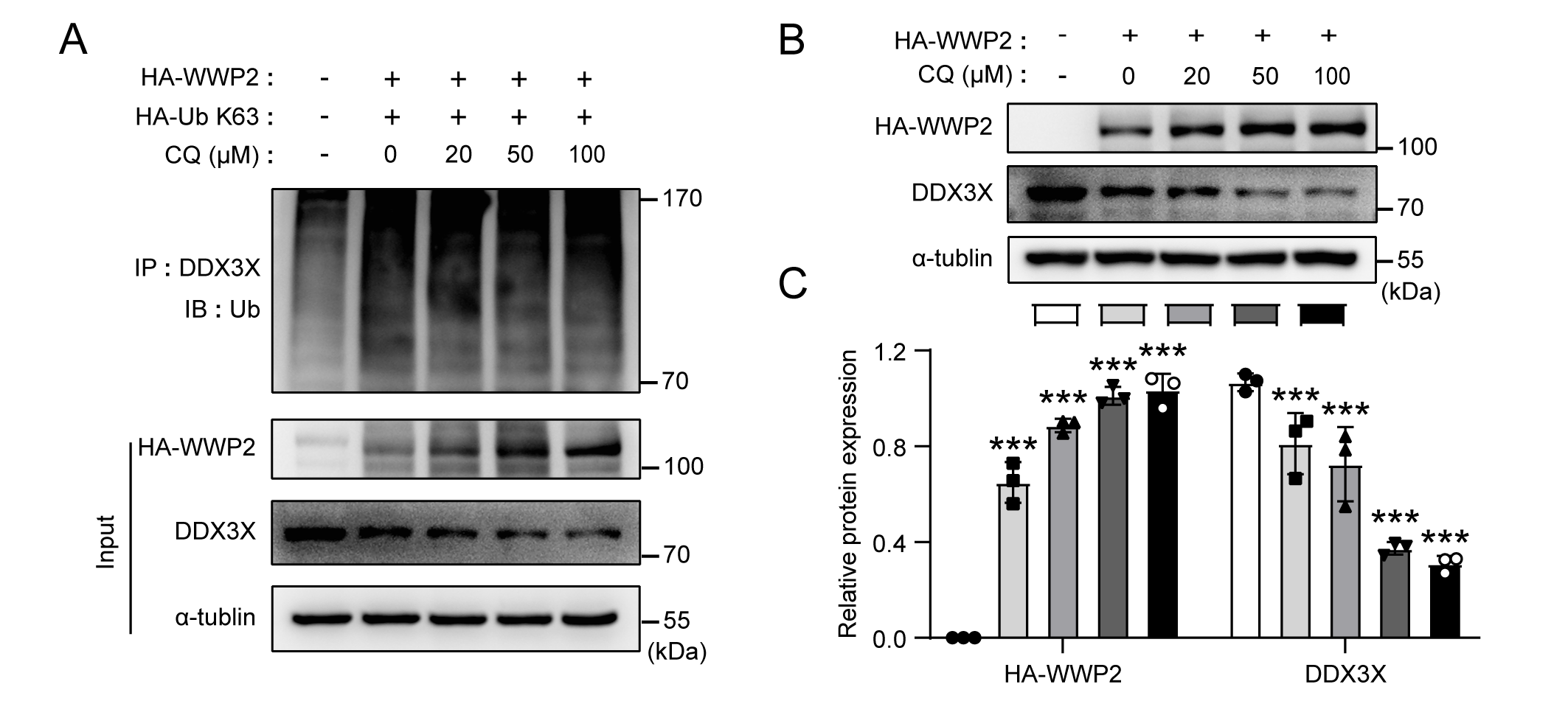


**Fig. S2 WWP2-mediated Ubiquitin (Ub)-dependent degradation of DDX3X is not a lysosome pathway.** A Effects of lysosome inhibitor chloroquine (CQ) on WWP2-mediated K63-linked polyubiquitination of DDX3X. Human umbilical vein endothelial cells (HUVECs) were transfected with expression plasmids encoding HA-WWP2 and Ub K63, and then treated with the indicated concentrations of CQ for 24 h or DMSO as control. **B, C** Representative Western blotting analysis (**B**) and quantitative analysis (**C**) of HA-WWP2 and DDX3X protein levels. HUVECs were transfected with expression plasmids encoding HA-WWP2, and then treated with the indicated concentrations of CQ for 24 h or DMSO as control. Values are shown as mean ± SD (***P < 0.001, one-way ANOVA with Dunnett’s multiple comparison post-hoc test).

**
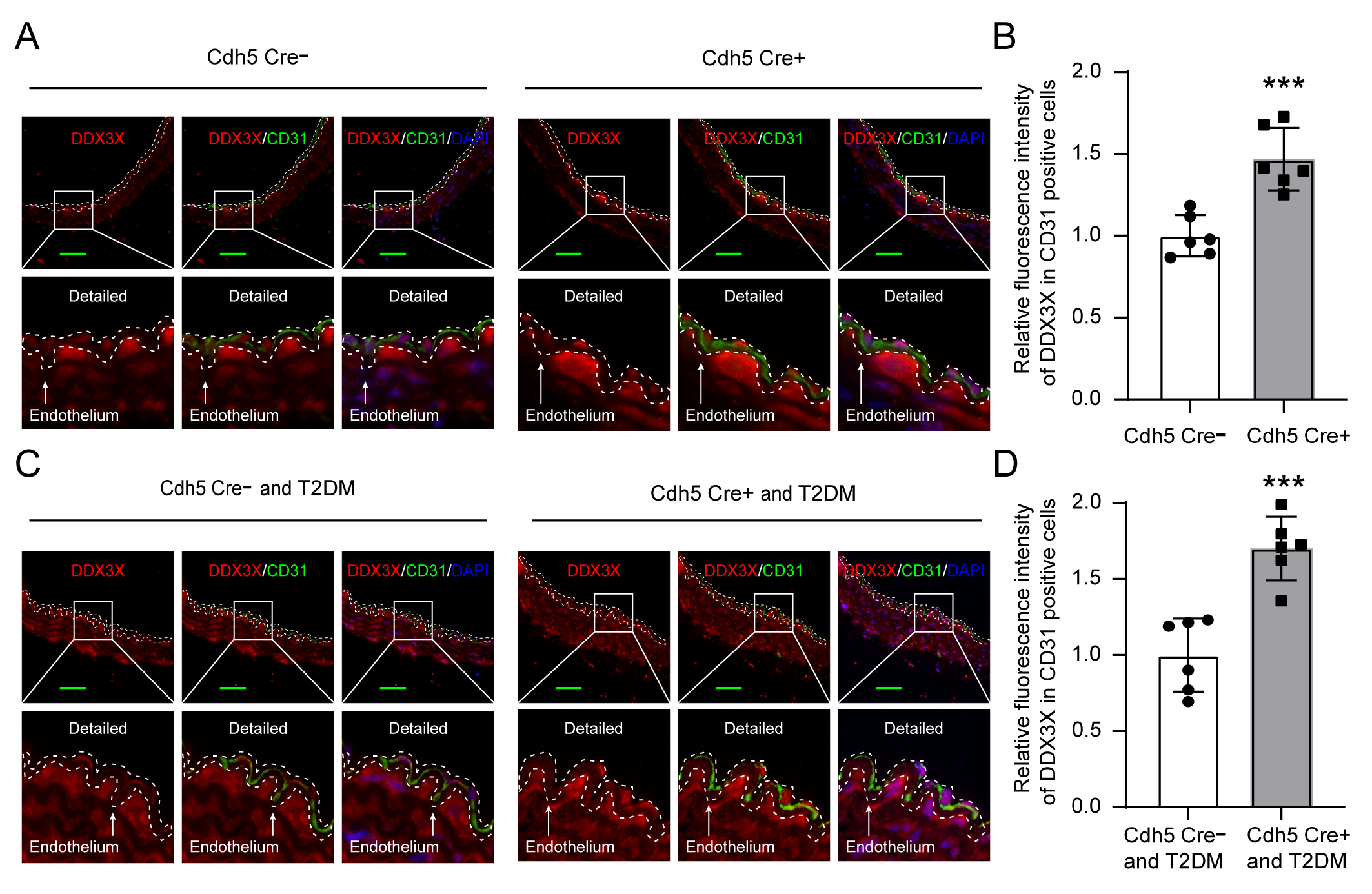
**

**Fig. S3 Endothelial-specific *Wwp2* knockout in mice leads to up-regulation of endothelial DDX3X expression. A, B, C, D** Representative immunofluorescent staining of aortic cross sections for the indicated mice (**A, C**) and quantitative analysis of relative fluorescence intensity of DDX3X in CD31 positive cells (**B, D**). (n = 6 mice per group). Red, DDX3X; Green, CD31 (a marker of ECs); blue, DAPI. scale bar 50 µm. (***P < 0.001, two-tailed unpaired Student t-tests). Cdh5 Cre-, (Cdh5 Cre-; *Wwp2^fl/fl^* mice); Cdh5 Cre+, (Cdh5 Cre+; *Wwp2^fl/fl^* mice); DAPI, 4′,6-diamidino-2-phenylindole; ECs, Endothelial cells; T2DM, Type 2 diabetes mellitus.

**Table S1. The clinical information of donors in the GSE156341 dataset.** BMI, Body Mass Index; HC, healthy control; T2DM, Type 2 diabetes mellitus.

| Donor ID | HbA1c | Gender | BMI | Age (years) | Other information |
| --- | --- | --- | --- | --- | --- |
| HC-1 | 5.4% | Male | 28.2 | 59 | Caucasian |
| HC-2 | 5.5% | Male | 21.0 | 28 | Hispanic |
| T2DM-1 | 9.5% | Female | 40.3 | 42 | Hispanic, T2DM > 10 years |
| T2DM-2 | 6.9% | Male | 45.8 | 46 | Hispanic, Untreated T2DM |

**Table S2. Cell types_top50_markers.** Cell types were identified according to the expression of top 50 known markers.

| gene | cluster | pct.1 | pct.2 | avg_log2FC | p_val | p_val_adj |
| --- | --- | --- | --- | --- | --- | --- |
| TRBC2 | 0 | 0.378 | 0.049 | 128.4874 | 5.38E-202 | 1.03E-197 |
| CYBA | 0 | 0.715 | 0.415 | 85.7703 | 1.35E-65 | 2.58E-61 |
| PLA2G2A | 0 | 0.198 | 0.009 | Inf | 2.47E-114 | 4.74E-110 |
| ADH1B | 0 | 0.191 | 0.006 | 169.6743 | 6.70E-113 | 1.28E-108 |
| COL6A2 | 0 | 0.198 | 0.014 | 113.8549 | 1.80E-106 | 3.46E-102 |
| MFAP5 | 0 | 0.206 | 0.036 | 201.6451 | 8.14E-87 | 1.56E-82 |
| FBLN1 | 0 | 0.175 | 0.006 | 215.8289 | 1.22E-102 | 2.34E-98 |
| IGFBP5 | 0 | 0.222 | 0.064 | 560.7405 | 1.18E-72 | 2.27E-68 |
| DCN | 0 | 0.221 | 0.065 | 818.6297 | 6.69E-73 | 1.28E-68 |
| LUM | 0 | 0.141 | 0.008 | 111.8858 | 1.39E-76 | 2.67E-72 |
| SERPINF1 | 0 | 0.221 | 0.093 | 176.5551 | 2.79E-44 | 5.35E-40 |
| CFD | 0 | 0.288 | 0.167 | 710.4568 | 4.47E-35 | 8.57E-31 |
| SFRP2 | 0 | 0.111 | 0.002 | 90.3995 | 3.56E-65 | 6.83E-61 |
| MT1G | 0 | 0.112 | 0.008 | 98.75587 | 2.67E-57 | 5.12E-53 |
| C3 | 0 | 0.175 | 0.074 | 186.7712 | 1.64E-36 | 3.15E-32 |
| AQP3 | 0 | 0.123 | 0.037 | 89.51636 | 3.86E-33 | 7.40E-29 |
| HTRA3 | 0 | 0.143 | 0.058 | 172.3894 | 2.26E-30 | 4.34E-26 |
| FKBP11 | 0 | 0.189 | 0.132 | 92.80601 | 4.93E-11 | 9.46E-07 |
| APOD | 0 | 0.159 | 0.106 | 335.3565 | 6.74E-13 | 1.29E-08 |
| COL1A2 | 0 | 0.167 | 0.116 | 120.3451 | 2.49E-12 | 4.77E-08 |
| COL6A1 | 0 | 0.171 | 0.167 | 108.5703 | 0.00867052 | 1 |
| RPS15 | 0 | 0.935 | 0.936 | 117.5096 | 3.29E-51 | 6.31E-47 |
| RPL8 | 0 | 0.908 | 0.948 | 114.6229 | 3.09E-146 | 5.91E-142 |
| ACTG1 | 0 | 0.866 | 0.924 | 222.8228 | 2.84E-89 | 5.44E-85 |
| PFN1 | 0 | 0.711 | 0.769 | 168.0039 | 6.90E-10 | 1.32E-05 |
| MT-ND3 | 0 | 0.897 | 0.959 | 90.09839 | 5.38E-273 | 1.03E-268 |
| XBP1 | 0 | 0.313 | 0.385 | 222.6757 | 1.49E-06 | 0.028621653 |
| GAPDH | 0 | 0.831 | 0.926 | 123.2804 | 4.23E-203 | 8.11E-199 |
| TAGLN | 0 | 0.135 | 0.246 | 91.54108 | 2.27E-29 | 4.36E-25 |
| IGFBP6 | 0 | 0.219 | 0.338 | 385.7795 | 9.38E-12 | 1.80E-07 |
| MT2A | 0 | 0.431 | 0.567 | 248.7948 | 3.54E-30 | 6.79E-26 |
| TXNIP | 0 | 0.702 | 0.843 | 258.892 | 1.64E-58 | 3.15E-54 |
| CCDC80 | 0 | 0.176 | 0.32 | 258.8639 | 4.38E-25 | 8.40E-21 |
| MT1E | 0 | 0.179 | 0.327 | 90.09481 | 9.94E-40 | 1.91E-35 |
| PPIB | 0 | 0.55 | 0.699 | 149.5736 | 5.00E-65 | 9.59E-61 |
| SSR4 | 0 | 0.569 | 0.718 | 216.6688 | 1.07E-76 | 2.05E-72 |
| SUB1 | 0 | 0.535 | 0.686 | 173.6184 | 1.86E-55 | 3.57E-51 |
| STMN1 | 0 | 0.174 | 0.337 | 110.2959 | 2.60E-53 | 4.99E-49 |
| ACKR3 | 0 | 0.123 | 0.304 | 102.3619 | 2.72E-67 | 5.22E-63 |
| SEC11C | 0 | 0.227 | 0.424 | 90.69073 | 9.13E-78 | 1.75E-73 |
| FSTL1 | 0 | 0.188 | 0.413 | 174.9189 | 2.22E-56 | 4.25E-52 |
| ATP1B3 | 0 | 0.234 | 0.464 | 95.83849 | 1.37E-94 | 2.62E-90 |
| GPX3 | 0 | 0.22 | 0.527 | 194.5405 | 4.59E-105 | 8.79E-101 |
| C1S | 0 | 0.169 | 0.495 | 113.5603 | 3.69E-146 | 7.08E-142 |
| HSP90B1 | 0 | 0.391 | 0.765 | 152.1356 | 2.65E-298 | 5.09E-294 |
| C1R | 0 | 0.179 | 0.566 | 156.4339 | 1.50E-203 | 2.88E-199 |
| GSN | 0 | 0.303 | 0.829 | 395.475 | 0 | 0 |
| TIMP1 | 0 | 0.312 | 0.839 | Inf | 0 | 0 |
| ADIRF | 0 | 0.073 | 0.619 | 528.486 | 0 | 0 |
| VWF | 1 | 0.916 | 0.025 | 136.9739 | 0 | 0 |
| ID1 | 1 | 0.945 | 0.066 | 79.26794 | 0 | 0 |
| EGFL7 | 1 | 0.92 | 0.044 | 87.90047 | 0 | 0 |
| SLC9A3R2 | 1 | 0.883 | 0.036 | 80.9307 | 0 | 0 |
| CAV1 | 1 | 0.884 | 0.043 | 119.6612 | 0 | 0 |
| SOX18 | 1 | 0.828 | 0.017 | 134.0168 | 0 | 0 |
| PECAM1 | 1 | 0.925 | 0.12 | 83.66144 | 0 | 0 |
| HSPG2 | 1 | 0.915 | 0.111 | 90.39426 | 0 | 0 |
| EPAS1 | 1 | 0.88 | 0.08 | 90.60134 | 0 | 0 |
| COL8A1 | 1 | 0.815 | 0.022 | 147.0725 | 0 | 0 |
| CLDN5 | 1 | 0.804 | 0.012 | 106.4273 | 0 | 0 |
| HYAL2 | 1 | 0.828 | 0.053 | 73.37353 | 0 | 0 |
| AQP1 | 1 | 0.782 | 0.018 | 71.59057 | 0 | 0 |
| HEG1 | 1 | 0.846 | 0.083 | 87.92195 | 0 | 0 |
| CLU | 1 | 0.917 | 0.155 | 92.25004 | 0 | 0 |
| IFI27 | 1 | 0.829 | 0.068 | 157.1712 | 0 | 0 |
| TM4SF1 | 1 | 0.738 | 0.013 | 102.1224 | 0 | 0 |
| ELN | 1 | 0.829 | 0.114 | 335.6132 | 0 | 0 |
| SPARCL1 | 1 | 0.816 | 0.16 | 87.92196 | 0 | 0 |
| CD59 | 1 | 0.87 | 0.218 | 85.03716 | 0 | 0 |
| SERPINE2 | 1 | 0.612 | 0.023 | 91.36912 | 0 | 0 |
| EMP1 | 1 | 0.668 | 0.088 | 69.09676 | 0 | 0 |
| AKR1C3 | 1 | 0.602 | 0.058 | 77.40269 | 0 | 0 |
| ADAMTS1 | 1 | 0.606 | 0.066 | 89.36468 | 0 | 0 |
| HSPB1 | 1 | 0.884 | 0.349 | 96.56782 | 0 | 0 |
| KLF2 | 1 | 0.862 | 0.336 | 80.90511 | 0 | 0 |
| IGFBP3 | 1 | 0.57 | 0.078 | 170.1557 | 0 | 0 |
| SELENOW | 1 | 0.846 | 0.407 | 83.5574 | 0 | 0 |
| SNHG7 | 1 | 0.75 | 0.311 | 77.51962 | 0 | 0 |
| SLPI | 1 | 0.581 | 0.152 | 477.4495 | 2.08E-285 | 3.99E-281 |
| FGL2 | 1 | 0.596 | 0.179 | 89.36465 | 1.98E-254 | 3.80E-250 |
| TNFSF10 | 1 | 0.67 | 0.267 | 138.4163 | 1.25E-282 | 2.40E-278 |
| ITLN1 | 1 | 0.399 | 0.012 | 484.6631 | 0 | 0 |
| CTGF | 1 | 0.433 | 0.057 | 337.5082 | 0 | 0 |
| NEAT1 | 1 | 0.891 | 0.516 | 132.6455 | 0 | 0 |
| CYR61 | 1 | 0.404 | 0.036 | 168.6414 | 0 | 0 |
| BST2 | 1 | 0.814 | 0.446 | 116.7649 | 0 | 0 |
| MMRN1 | 1 | 0.369 | 0.009 | 226.6484 | 0 | 0 |
| GJA4 | 1 | 0.278 | 0.003 | 68.09821 | 0 | 0 |
| ISG15 | 1 | 0.545 | 0.281 | 115.3317 | 5.46E-128 | 1.05E-123 |
| CX3CL1 | 1 | 0.263 | 0.002 | 82.10632 | 9.72E-301 | 1.86E-296 |
| HLA-E | 1 | 0.942 | 0.766 | 113.8877 | 0 | 0 |
| SRP14 | 1 | 0.889 | 0.742 | 70.78704 | 4.85E-221 | 9.30E-217 |
| ACKR1 | 1 | 0.103 | 0.005 | 104.978 | 4.57E-93 | 8.75E-89 |
| VIM | 1 | 0.988 | 0.894 | 206.2491 | 3.02E-305 | 5.79E-301 |
| MT-ND4 | 1 | 0.939 | 0.88 | 112.4477 | 1.56E-155 | 3.00E-151 |
| RPL3 | 1 | 0.964 | 0.943 | 119.6612 | 1.08E-24 | 2.07E-20 |
| TPT1 | 1 | 0.99 | 0.986 | 158.614 | 2.05E-12 | 3.94E-08 |
| EEF1A1 | 1 | 0.992 | 0.988 | 82.14166 | 6.42E-24 | 1.23E-19 |
| RPS2 | 1 | 0.927 | 0.967 | 67.69888 | 1.38E-10 | 2.64E-06 |
| LYZ | 2 | 0.984 | 0.116 | 399.1469 | 0 | 0 |
| CD14 | 2 | 0.976 | 0.12 | 130.441 | 0 | 0 |
| FCER1G | 2 | 0.993 | 0.18 | 86.05539 | 0 | 0 |
| AIF1 | 2 | 0.989 | 0.178 | 94.57221 | 0 | 0 |
| CD163 | 2 | 0.902 | 0.095 | 89.2717 | 0 | 0 |
| MAFB | 2 | 0.927 | 0.134 | 134.6996 | 0 | 0 |
| MS4A4A | 2 | 0.891 | 0.102 | 94.23633 | 0 | 0 |
| TYROBP | 2 | 0.996 | 0.211 | 146.4891 | 0 | 0 |
| S100A9 | 2 | 0.857 | 0.096 | 634.3063 | 0 | 0 |
| HMOX1 | 2 | 0.829 | 0.082 | 116.3246 | 0 | 0 |
| VSIG4 | 2 | 0.849 | 0.111 | 116.3591 | 0 | 0 |
| CPM | 2 | 0.764 | 0.044 | 107.3209 | 0 | 0 |
| C1QA | 2 | 0.882 | 0.204 | 302.4864 | 0 | 0 |
| C1QC | 2 | 0.831 | 0.163 | 549.004 | 0 | 0 |
| CTSS | 2 | 0.998 | 0.344 | 99.02844 | 0 | 0 |
| C1QB | 2 | 0.849 | 0.205 | 396.2616 | 1.22E-299 | 2.33E-295 |
| HLA-DQA1 | 2 | 0.766 | 0.16 | 159.6596 | 6.15E-281 | 1.18E-276 |
| CTSB | 2 | 0.984 | 0.378 | 111.7545 | 3.58E-306 | 6.85E-302 |
| CTSZ | 2 | 0.973 | 0.391 | 91.84899 | 5.97E-263 | 1.14E-258 |
| HLA-DRA | 2 | 0.98 | 0.436 | 641.9624 | 3.16E-259 | 6.05E-255 |
| FOS | 2 | 0.944 | 0.414 | 87.50812 | 3.68E-209 | 7.05E-205 |
| PSAP | 2 | 0.996 | 0.484 | 162.4982 | 4.74E-243 | 9.08E-239 |
| LGMN | 2 | 0.61 | 0.116 | 93.9359 | 1.48E-209 | 2.84E-205 |
| CTSD | 2 | 0.971 | 0.481 | 131.2576 | 2.18E-229 | 4.18E-225 |
| HLA-DPA1 | 2 | 0.949 | 0.484 | 390.4811 | 3.41E-223 | 6.53E-219 |
| PLTP | 2 | 0.695 | 0.232 | 222.6596 | 2.03E-141 | 3.90E-137 |
| HLA-DRB1 | 2 | 0.98 | 0.521 | 633.8543 | 2.67E-240 | 5.11E-236 |
| CCL3 | 2 | 0.512 | 0.062 | 200.9942 | 4.01E-240 | 7.69E-236 |
| HLA-DPB1 | 2 | 0.938 | 0.494 | 217.3674 | 3.56E-214 | 6.83E-210 |
| SAT1 | 2 | 0.987 | 0.558 | 152.4377 | 2.49E-266 | 4.76E-262 |
| S100A8 | 2 | 0.441 | 0.024 | 204.3831 | 0 | 0 |
| CST3 | 2 | 0.996 | 0.596 | 106.2799 | 4.13E-257 | 7.93E-253 |
| CD74 | 2 | 0.998 | 0.606 | Inf | 1.37E-234 | 2.63E-230 |
| IER2 | 2 | 0.829 | 0.5 | 113.4934 | 6.74E-79 | 1.29E-74 |
| APOC1 | 2 | 0.396 | 0.069 | 165.4304 | 9.24E-131 | 1.77E-126 |
| SELENOP | 2 | 0.706 | 0.386 | 323.9316 | 6.84E-106 | 1.31E-101 |
| APOE | 2 | 0.428 | 0.109 | 370.2931 | 5.01E-97 | 9.60E-93 |
| FCGBP | 2 | 0.336 | 0.018 | 112.0485 | 1.49E-256 | 2.86E-252 |
| HLA-DRB5 | 2 | 0.428 | 0.13 | 181.3 | 5.38E-71 | 1.03E-66 |
| RNASE1 | 2 | 0.637 | 0.343 | 80.31137 | 3.14E-68 | 6.02E-64 |
| IL1R2 | 2 | 0.287 | 0.01 | 77.71044 | 4.92E-265 | 9.43E-261 |
| FCN1 | 2 | 0.278 | 0.002 | 86.39143 | 0 | 0 |
| IL1B | 2 | 0.267 | 0.005 | 164.2556 | 1.01E-297 | 1.94E-293 |
| GPNMB | 2 | 0.325 | 0.083 | 100.2922 | 9.92E-64 | 1.90E-59 |
| HIST1H4C | 2 | 0.755 | 0.521 | 87.34173 | 9.29E-28 | 1.78E-23 |
| SH3BGRL3 | 2 | 0.987 | 0.765 | 84.45633 | 5.24E-117 | 1.01E-112 |
| CCL4 | 2 | 0.301 | 0.158 | 295.2728 | 4.20E-18 | 8.06E-14 |
| CCL4L2 | 2 | 0.134 | 0.025 | 100.5091 | 2.04E-37 | 3.91E-33 |
| MT-CO1 | 2 | 1 | 0.921 | 188.5135 | 1.15E-202 | 2.21E-198 |
| TMSB10 | 2 | 0.998 | 0.942 | 277.5086 | 3.44E-141 | 6.60E-137 |
| MS4A1 | 3 | 0.938 | 0.022 | 23.98517 | 0 | 0 |
| CD79B | 3 | 0.897 | 0.091 | 9.780559 | 2.41E-237 | 4.61E-233 |
| BANK1 | 3 | 0.759 | 0.005 | 5.293513 | 0 | 0 |
| VPREB3 | 3 | 0.724 | 0.002 | 7.973731 | 0 | 0 |
| LINC00926 | 3 | 0.717 | 0.006 | 4.357253 | 0 | 0 |
| RALGPS2 | 3 | 0.71 | 0.041 | 3.84422 | 5.41E-279 | 1.04E-274 |
| IGHD | 3 | 0.648 | 0.001 | 13.11456 | 0 | 0 |
| CD37 | 3 | 0.979 | 0.342 | 27.40931 | 2.01E-110 | 3.85E-106 |
| FCER2 | 3 | 0.621 | 0.001 | 6.009123 | 0 | 0 |
| FCMR | 3 | 0.697 | 0.081 | 4.174698 | 2.97E-149 | 5.69E-145 |
| HVCN1 | 3 | 0.669 | 0.087 | 3.536386 | 2.09E-128 | 4.01E-124 |
| ARHGAP24 | 3 | 0.607 | 0.041 | 2.555908 | 8.42E-200 | 1.61E-195 |
| CD22 | 3 | 0.51 | 0.003 | 3.733742 | 0 | 0 |
| BLK | 3 | 0.497 | 0.001 | 2.187789 | 0 | 0 |
| FCRLA | 3 | 0.497 | 0.002 | 4.516842 | 0 | 0 |
| BCL11A | 3 | 0.497 | 0.007 | 1.496034 | 0 | 0 |
| TNFRSF13C | 3 | 0.476 | 0.004 | 2.985221 | 0 | 0 |
| TSTD1 | 3 | 0.683 | 0.219 | 2.618102 | 6.90E-42 | 1.32E-37 |
| RASGRP2 | 3 | 0.559 | 0.129 | 4.935598 | 2.76E-52 | 5.29E-48 |
| CD19 | 3 | 0.421 | 0.004 | 2.01596 | 0 | 0 |
| LINC02397 | 3 | 0.414 | 0.003 | 2.017451 | 0 | 0 |
| RHOH | 3 | 0.545 | 0.135 | 1.621947 | 9.57E-44 | 1.83E-39 |
| FCRL1 | 3 | 0.386 | 0 | 1.718362 | 0 | 0 |
| CLEC2D | 3 | 0.566 | 0.191 | 1.421086 | 8.74E-30 | 1.68E-25 |
| CD24 | 3 | 0.352 | 0.004 | 3.224137 | 0 | 0 |
| AFF3 | 3 | 0.359 | 0.011 | 1.149609 | 6.01E-206 | 1.15E-201 |
| RUBCNL | 3 | 0.345 | 0.016 | 1.246339 | 6.06E-150 | 1.16E-145 |
| RPL17 | 3 | 0.738 | 0.415 | 1.122459 | 2.62E-17 | 5.03E-13 |
| TNFAIP8 | 3 | 0.586 | 0.264 | 1.865513 | 1.47E-18 | 2.81E-14 |
| GAPT | 3 | 0.338 | 0.022 | 1.566271 | 1.50E-114 | 2.87E-110 |
| IL4R | 3 | 0.469 | 0.163 | 2.183589 | 1.25E-25 | 2.39E-21 |
| LINC01857 | 3 | 0.297 | 0.003 | 3.554611 | 3.05E-292 | 5.84E-288 |
| FCRL2 | 3 | 0.29 | 0.002 | 3.185811 | 0 | 0 |
| FAM129C | 3 | 0.269 | 0.001 | 1.106253 | 0 | 0 |
| BACH2 | 3 | 0.269 | 0.025 | 1.080287 | 4.25E-66 | 8.14E-62 |
| TLR10 | 3 | 0.248 | 0.005 | 2.511716 | 2.45E-193 | 4.70E-189 |
| ZCCHC7 | 3 | 0.366 | 0.124 | 1.087479 | 1.08E-17 | 2.06E-13 |
| PLEKHF2 | 3 | 0.324 | 0.087 | 1.925763 | 1.03E-22 | 1.98E-18 |
| TMEM154 | 3 | 0.31 | 0.074 | 1.994721 | 5.07E-26 | 9.72E-22 |
| STRBP | 3 | 0.303 | 0.068 | 1.484598 | 1.33E-27 | 2.54E-23 |
| MDM4 | 3 | 0.552 | 0.326 | 1.591866 | 1.45E-09 | 2.77E-05 |
| CD200 | 3 | 0.221 | 0.013 | 1.091675 | 4.19E-81 | 8.04E-77 |
| STAG3 | 3 | 0.221 | 0.02 | 5.807484 | 1.74E-56 | 3.33E-52 |
| ORAI2 | 3 | 0.283 | 0.09 | 1.763306 | 2.47E-15 | 4.74E-11 |
| IFT57 | 3 | 0.297 | 0.149 | 1.542622 | 1.47E-07 | 0.002815267 |
| KCNG1 | 3 | 0.145 | 0 | 1.152941 | 6.59E-216 | 1.26E-211 |
| DGKD | 3 | 0.221 | 0.085 | 1.351458 | 5.15E-09 | 9.88E-05 |
| AIM2 | 3 | 0.131 | 0.016 | 2.111459 | 3.48E-25 | 6.66E-21 |
| LINC01781 | 3 | 0.11 | 0.002 | 3.734479 | 4.96E-89 | 9.51E-85 |
| POLR2J3.1 | 3 | 0.214 | 0.321 | 1.271674 | 0.00346159 | 1 |

**Table S3. Expression changes of genes in KEGG apoptosis pathway.** NS, not significant.

| Genes | log2FoldChange | Up or Down | pvalue |
| --- | --- | --- | --- |
| TNFRSF1A | -11.4711 | Down | 6.06E-05 |
| ENDOD1 | -6.39456 | Down | 0.004015 |
| IRAK3 | -4.83697 | Down | 5.31E-16 |
| AKT1 | -1.62638 | Down | 0.012246 |
| IRAK1 | -0.84262 | Down | 0.00025 |
| TP53 | -0.40472 | Down | 5.21E-07 |
| PRKAR1B | -0.29559 | Down | 0.003707 |
| MAP3K14 | -0.27865 | Down | 1.36E-05 |
| CASP10 | -0.25229 | Down | 4.79E-05 |
| PPP3R1 | -0.17589 | Down | 0.00185 |
| AKT2 | -0.06329 | NS | 0.796737 |
| APAF1 | 0.033337 | NS | 0.306068 |
| BAX | -3.71901 | NS | 0.746106 |
| BCL2L1 | -1.28284 | NS | 0.109617 |
| BIRC2 | 0.075307 | NS | 0.3128 |
| BIRC3 | -8.17258 | NS | 0.659759 |
| CAPN2 | -0.00183 | NS | 0.387252 |
| CASP9 | 0.015831 | NS | 0.750752 |
| CHUK | 0.009169 | NS | 0.397545 |
| DFFA | 0.180767 | NS | 0.056924 |
| DFFB | 0.034946 | NS | 0.073306 |
| FADD | 0.009464 | NS | 0.559189 |
| FASLG | 0.003038 | NS | 0.240666 |
| IKBKG | -0.02376 | NS | 0.543958 |
| IL1A | 0.003038 | NS | 0.240666 |
| IL1B | 0.006071 | NS | 0.096814 |
| IL1RAP | 0.052645 | NS | 0.050182 |
| IRAK2 | 0.035577 | NS | 0.832912 |
| MYD88 | 0.013979 | NS | 0.648343 |
| NGFR | 0.006071 | NS | 0.096814 |
| PIK3CD | 0.007373 | NS | 0.945023 |
| PIK3CG | -0.00977 | NS | 0.306074 |
| PIK3R2 | -0.02445 | NS | 0.065991 |
| PIK3R5 | 0.006071 | NS | 0.096814 |
| PRKACA | -0.01407 | NS | 0.450751 |
| PRKAR2B | 0.003867 | NS | 0.387445 |
| RELA | -0.10871 | NS | 0.185764 |
| RIPK1 | 0.030195 | NS | 0.694875 |
| TNF | 0.003867 | NS | 0.387445 |
| TNFRSF10B | -0.09279 | NS | 0.967331 |
| TNFRSF10C | 0.009582 | NS | 0.360312 |
| TRAF2 | 0.067382 | NS | 0.059103 |
| XIAP | 0.270839 | NS | 0.062127 |
| BCL2 | 0.044116 | Up | 0.001054 |
| CASP6 | 0.058287 | Up | 0.01188 |
| BID | 0.085242 | Up | 0.005185 |
| CASP8 | 0.090806 | Up | 0.014987 |
| PPP3CC | 0.113839 | Up | 0.022178 |
| AIFM1 | 0.128435 | Up | 0.007661 |
| CASP7 | 0.139411 | Up | 0.000182 |
| TRADD | 0.16516 | Up | 0.000449 |
| EXOG | 0.169053 | Up | 6.57E-07 |
| FAS | 0.209538 | Up | 0.000172 |
| PPP3CB | 0.214466 | Up | 0.009912 |
| TNFRSF10A | 0.220595 | Up | 0.000629 |
| CAPN1 | 0.229469 | Up | 0.000304 |
| IKBKB | 0.235889 | Up | 0.020452 |
| PRKACB | 0.257188 | Up | 0.000158 |
| ENDOG | 0.260837 | Up | 2.47E-07 |
| IRAK4 | 0.269949 | Up | 0.000139 |
| CASP3 | 0.291562 | Up | 1.36E-06 |
| NFKB1 | 0.309986 | Up | 0.005233 |
| BAD | 0.31235 | Up | 3.25E-05 |
| PIK3CB | 0.362508 | Up | 4.16E-06 |
| PRKAR2A | 0.490366 | Up | 0.001296 |
| PIK3CA | 0.49137 | Up | 0.000024 |
| PPP3CA | 0.737714 | Up | 5.57E-08 |
| CSF2RB | 0.993766 | Up | 2.74E-09 |
| PRKX | 1.153015 | Up | 1.25E-11 |
| CYCS | 1.211269 | Up | 0.001686 |
| ATM | 1.26837 | Up | 1.49E-09 |
| PIK3R1 | 1.304442 | Up | 2.53E-09 |
| PRKAR1A | 1.318112 | Up | 0.000296 |
| CFLAR | 1.429597 | Up | 8.42E-10 |
| NFKBIA | 1.456484 | Up | 1.91E-26 |
| CHP1 | 1.488709 | Up | 9.87E-08 |
| IL3RA | 1.603713 | Up | 1.21E-71 |
| TNFRSF10D | 2.591713 | Up | 2.59E-44 |
| AKT3 | 2.842625 | Up | 9.65E-34 |
| IL1R1 | 3.21508 | Up | 4.36E-05 |
| PIK3R3 | 18.99724 | Up | 1.8E-08 |
| TNFSF10 | 85.58278 | Up | 5.99E-17 |
